# A dynamically coherent pattern of rhythms that matches between distant species across the evolutionary scale

**DOI:** 10.1038/s41598-023-32286-0

**Published:** 2023-04-01

**Authors:** J. M. Kembro, A. G. Flesia, P. S. Nieto, J. M. Caliva, D. Lloyd, S. Cortassa, M. A. Aon

**Affiliations:** 1 Instituto de Investigaciones Biológicas y Tecnológicas, Consejo Nacional de Investigaciones Científicas y Técnicas, Universidad Nacional de Córdoba, Córdoba, Argentina; 2 Facultad de Ciencias Exactas, Físicas y Naturales, Instituto de Ciencia y Tecnología de los Alimentos (ICTA), Universidad Nacional de Córdoba, Córdoba, Argentina; 3 Universidad Nacional de Córdoba, Facultad de Ciencias Exactas, Físicas y Naturales, Departamento de Química, Cátedra de Química Biológica, Córdoba, Argentina; 4 Facultad de Matemática, Astronomía, Física y Computación, Consejo Nacional de Investigaciones Científicas y Técnicas (CONICET), Centro de Investigación y Estudios de La Matemática (CIEM, CONICET-UNC), Universidad Nacional de Córdoba, Córdoba, Argentina; 5 Facultad de Matemática, Astronomía, Física y Computación, Consejo Nacional de Investigaciones Científicas y Técnicas (CONICET), Instituto de Física Enrique Gaviola (IFEG, CONICET-UNC), Universidad Nacional de Córdoba, Córdoba, Argentina; 6 Schools of Bioscience and Engineering, Cardiff University, Cardiff, Wales, UK; 7 Laboratory of Cardiovascular Science, National Institute on Aging, NIH, Baltimore, MD USA; 8 Translational Gerontology Branch, National Institute on Aging, NIH, Baltimore, MD USA

**Keywords:** Computational biology and bioinformatics, Systems biology

## Abstract

We address the temporal organization of circadian and ultradian rhythms, crucial for understanding biological timekeeping in behavior, physiology, metabolism, and alignment with geophysical time. Using a newly developed five-steps wavelet-based approach to analyze high-resolution time series of metabolism in yeast cultures and spontaneous movement, metabolism, and feeding behavior in mice, rats, and quails, we describe a dynamically coherent pattern of rhythms spanning over a broad range of temporal scales (hours to minutes). The dynamic pattern found shares key features among the four, evolutionary distant, species analyzed. Specifically, a branching appearance given by splitting periods from 24 h into 12 h, 8 h and below in mammalian and avian species, or from 14 h down to 0.07 h in yeast. Scale-free fluctuations with long-range correlations prevail below ~ 4 h. Synthetic time series modeling support a scenario of coexisting behavioral rhythms, with circadian and ultradian rhythms at the center of the emergent pattern observed.

## Introduction

Biological timekeeping associated with daily profound variations in, e.g., light, temperature, is crucial for adaptation and survival of living organisms. Circadian rhythms with a period of about a day were first described in 1729 by the French astronomer de Mairan, and since then extensively studied and shown to be ubiquitous from protists to humans^1–5^. The discovery of the molecular mechanisms by which organisms anticipate and adapt to daily environmental cues through clock genes that control circadian oscillations in cells and tissues, led to the 2017 Nobel Prize in Physiology or Medicine^6^. Ultradian rhythms with periods shorter than 24 h have also been reported in a broad variety of organisms^3,7,8^ but the study of their origin and functional role lags far behind the dominant circadian research.

Frequency- and phase-coordination exists between different types of rhythms. Evidence suggests that signals from the suprachiasmatic nucleus (SCN) might synchronize in avian^9^ and mammalian^2^ (including humans) peripheral circadian clocks^10^. In mammals, including humans, existing knowledge shows that both the liver clock and feeding rhythms are also required for temporal coordination and alignment of physiology and metabolism with geophysical time^11^. Moreover, peripheral tissues can be directly entrained in response to environmental signals without the need for SCN intervention^12^. For example, feeding cycles can entrain the liver independently of the SCN and the light cycle^13^. Circadian gene expression in response to food-induced phase resetting has also been observed in cells from kidney, heart, and pancreas^14^. In addition to 24 h transcriptional rhythms, the liver and other tissues express ultradian rhythms with period lengths of 12, 8 and 6 h^8,15,16^. Specifically, recent studies have proposed that the mammalian 12-h rhythm is not only cell autonomous, but that can be linked to a dedicated “12-h clock”, separate from the circadian clock, that also functions to coordinate cellular stress with metabolism^7,17,18^.

Biological systems are spatially and temporally distributed, built from a dynamic web of interconnected feedback loops marked by interdependence, pleiotropy, and redundancy^19^. These features challenge our ability to extract fundamental quantitative information from their dynamics. For instance, frequency relations are observed between rhythms, with more frequent rhythms exhibiting frequencies that can be described by multiples of less frequent ones^20^. A combination of different methods for detecting rhythms appears to be the most reliable approach^21–23^ since the majority of the wide variety of methods employed for chronobiological analysis, e.g., Fourier, Autocorrelation, Extended cosinor, Maximum Entropy Spectral Analysis (MESA), Enright and Lomb–Scargle periodograms, eigenvalue/pencil method, multiple signal classification (MUSIC)^24–28^, all assume that the oscillatory processes under study are stationary (i.e. mean and variance does not change over time) while a priori information about their stationarity and the presence of noise in the time series is not always possible^29^. Methodological approximations for quantification of irregular and or nonstationary rhythms have been studied through Singular Spectrum Analysis (SSA)^22^ as well as wavelet based analysis^21,30^. For example, in non-stationary avian locomotion time series, wavelet analysis enabled unambiguous and highly sensitive detection of ultradian rhythms, even with short periods (< 1–2 h) on an individual animal level, while traditional analytical tools such as Power Spectrum and Enright’s based analyses exhibited limitations in their detection, notable for time scales below daily rhythms^23,31^.

Given the evident multiplicity of existing rhythms, an important question concerns their coordination in distinct temporal domains. More precisely, how circadian and ultradian rhythms can be dynamically orchestrated? It is difficult to overstate the importance of these questions, since rhythmic misalignment is linked to aging and disease^32–35^ as well as their mechanistic underpinnings with physiology, metabolism^36^ and behavior^31^.

The living state can be understood as homeodynamic^37^ rather than homeostatic^38^, and non-linearity of functional activities lead to an emergent plethora of oscillatory, rhythmic and timed outputs^37,39,40^. Advanced signal processing analysis of experimentally obtained time series from self-synchronized *Saccharomyces cerevisiae* (yeast) continuous culture^41^, mammalian cardiac cells^42,43^, quail locomotion^23^ and mice wheel running^21^ have shown multi-oscillatory, dynamically functional patterns of behavior. These dynamic patterns hold over a broad range of temporal scales for at least 3 orders of magnitude: ~ 13 h–4 min in yeast (dissolved O_2_ and CO_2_)^41,44^, 100 s–220 ms in cardiac muscle cells (mitochondrial membrane potential and NAD(P)H)^42,44^, 24 h–15 min in quails locomotion^21,23,31^. However, none of these prior studies assessed coherence between rhythms associated with different biological processes operating at different time scales within the same model organism.

It is not surprising that important insights on multi-oscillatory dynamics were obtained from yeast^45–47^. Most of our basic understanding of networks from central metabolism has come from research in this species since the nineteenth century^48,49^. Although *S. cerevisiae* is evolutionarily separated by ~ 1.5 billion years from mammalian cells^50^ and has about fivefold fewer genes that humans (^51^; reviewed in ^52^), it has provided fundamental understanding of molecular function, deficiencies, and disorders in mammals and other species^45–47^. Despite the wide evolutionary time separation and number of genes, human gene orthologs are able to complement growth defects in nearly half of yeast genes (43%)^53^. Ease of gene replacement was best predicted by properties of specific gene modules (i.e., proteins in the same pathway or complex) rather than sequence similarity. This modularity of gene replacement suggests that ancestral essential genes are critically retained in pathway-specific manner and resilient to drift in sequence, further highlighting the usefulness of yeast as an experimental model in the understanding of basic biological mechanisms^53^.

Previous comparative work between the dynamics of time series obtained from yeast and isolated cardiac cells data has been explored, detecting similarities between them^50^. However, due to temporal span limitations regarding data acquisition in cardiac cells, the study was limited to the milliseconds to few minutes time span^44^. More recently, publicly available datasets offer the opportunity to not only compare long, high resolution, time series among evolutionarily distant species, but also coherence among distinct rhythms within species. These datasets include mice and rat time series from metabolic cages^54^, activity and feeding behavior in C57BL/6 mice^55,56^ and the high-resolution locomotor, feeding and drinking time series from Japanese quails^23^.

Herein, we address the general question of organization and coordination between rhythms at the organism level, by studying high resolution time series spanning broad temporal scales, from a few minutes to several days. Time series from living systems are predominantly nonstationary since they change over time, frequently in a rhythmic fashion, such as, e.g., animal physiology, metabolism, and behavior involving movement, feeding, mating or in synchronized cellular systems exhibiting oscillatory metabolic and gene expression dynamics^25^. We utilize an advanced integrative methodological approach (GaMoSEC) for analyzing extensive time series obtained from evolutionarily distant species such as yeast (*S. cerevisiae*), mammal (*Mus musculus, Rattus norvegicus*) and avian (*Coturnix japonica*). With our set of newly developed analytical tools based on wavelet analyses^21^ we explore, detect, identify and compare rhythms in the circadian and ultradian temporal domains. We found an overall emergent dynamic pattern of rhythms, shared amongst the evolutionarily distant species studied, and both in mammalian and avian instances circadian and ultradian temporal domains, in agreement with its potential universality.

## Results

To investigate the temporal organization of rhythms in circadian and ultradian domains, we comprehensively address the analysis of nonstationary time series from distinct and widely studied organisms such as yeast, mouse, rat and quail. We utilize an integrated methodological approach (GaMoSEC), comprising a wide range of tools based on wavelet analyses, which is applied in five steps: Gaussian and complex Morlet wavelets, Synchrosqueezing, Empirical Wavelet Decomposition (EWD) and wavelet Coherence^21^.

### Time series of yeast metabolism in entrained and/or spontaneously synchronous continuous culture.

**Figure 1:**
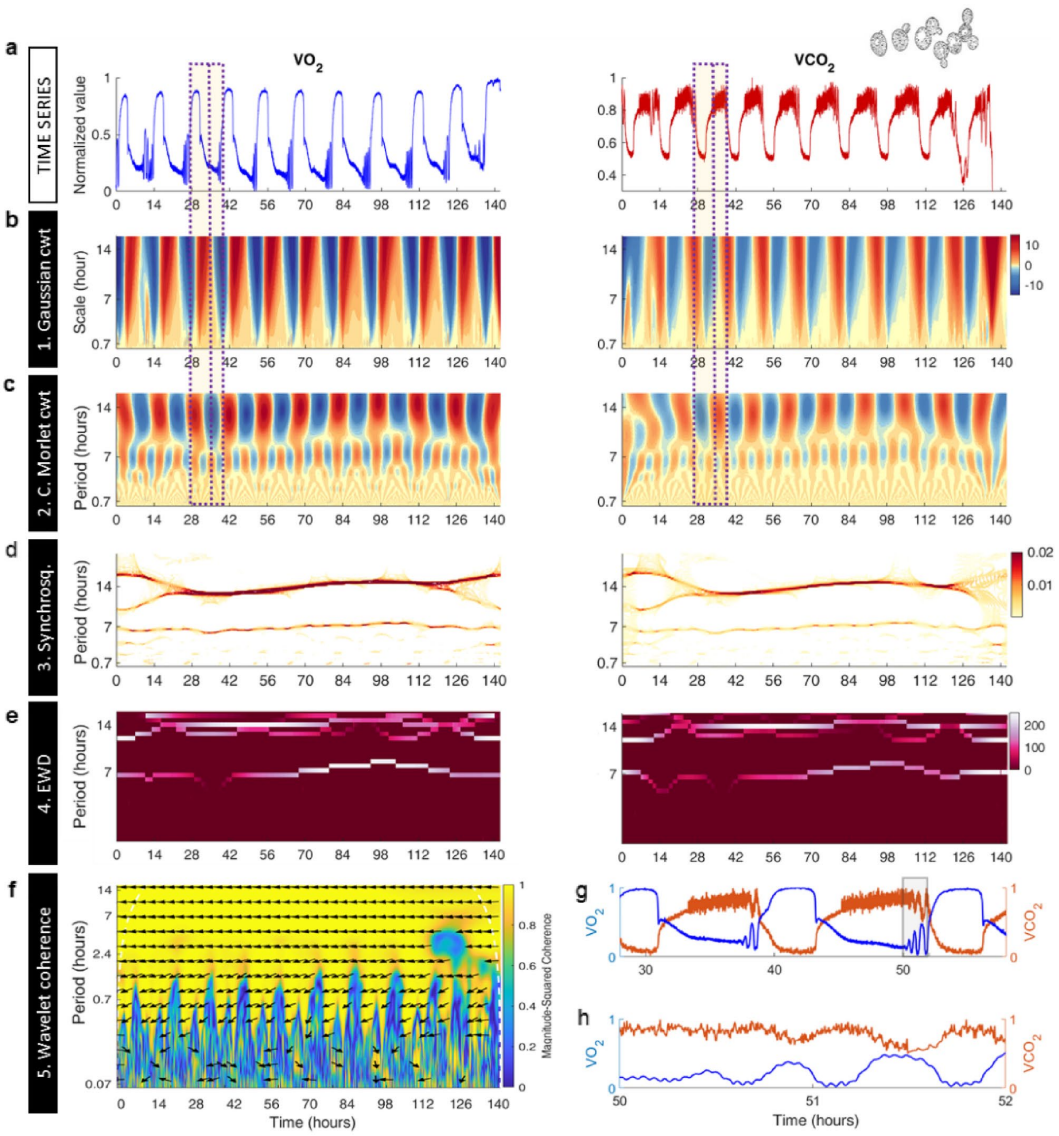
The 5-steps of GaMoSEC analysis were applied to time series of O_2_ and CO_2_ signals obtained by membrane-inlet mass spectrometry (MIMS) from oscillating continuous cultures of *S. cerevisiae*. (**a**) Relative MIMS signals of the m/z = 32 and 44 components versus time, corresponding to the O_2_ (blue) and CO_2_ (red) signals, respectively. Time is given in hours after the start of the continuous operation. Yeast continuous cultures were operated as described under Methods at a total volume of 800 ml; medium flow rate, 1 ml/min, i.e., dilution rate D = 0.0765 h^−1^. From this visualization it is apparent that the large-amplitude oscillation shows substantial cycle-to-cycle variability, with cycle times of 11.7–15.5 h, giving a mean of 13.66 h (SD, n = 8)^44^. (**b**) Analysis of the time series shown in “a” with the Gaussian cwt. This wavelet highlights variability and transitions between states at a given time scale. Note that the principal oscillation is observed in red orange over a broad range of scales. Fluctuations are visible for shorter time scales (≤ 7 h). (**c**) Analysis of the time series shown in “a” with the complex Morlet cwt, only the real part is shown. Note the bifurcation-like pattern marking the different oscillations that compose the signal. (**d**) Synchrosqueezing method applied to time series shown in “a”. Dark  horizontal bands indicate the estimated period of the signal at the time scales around 13.6 and 6.8 h. Note a slight increase in period over time in the ~ 14 h range, as previously mentioned in^44^. This period is longer than the mean doubling time of 9 h (= ln2/D), as discussed elsewhere^41^. The biological bases for all three oscillatory outputs of the yeast culture have been confirmed by exclusion of the possible influences of variations of aeration or stirring, pulsed medium addition, cycles of NaOH addition and pH variation, or cycles of temperature control^41,58^. (**e**) Empirical Wavelet Decomposition applied to the time series shown in “a”. Note that since the period changes over time, at the time scale around 13.6 h, this method shows a mixture of different horizontal lines, which is not observed for scales around 6.8 h. (**f**) Wavelet coherence analysis was performed between the two-time series shown in Fig. 1a. Color-scale represents the magnitude squared coherence between O_2_ and CO_2_ for a given time scale. Note high positive values for scales up to ~ 0.07 h (~ 4 min). Arrows indicate phase relationships between signals at a given time scale. The 180º angle indicates an antiphase activity, as noticeable in the time series shown in A, for both large and shorter time scales. (**g**–**h**) Zoom in on time series  depicted in “a” showing the antiphase relationship between them at both, large (13.6 h) and few minutes temporal scales. Image yeast: https://commons.wikimedia.org/wiki/File:Yeast_(PSF).png.

**Figure 2:**
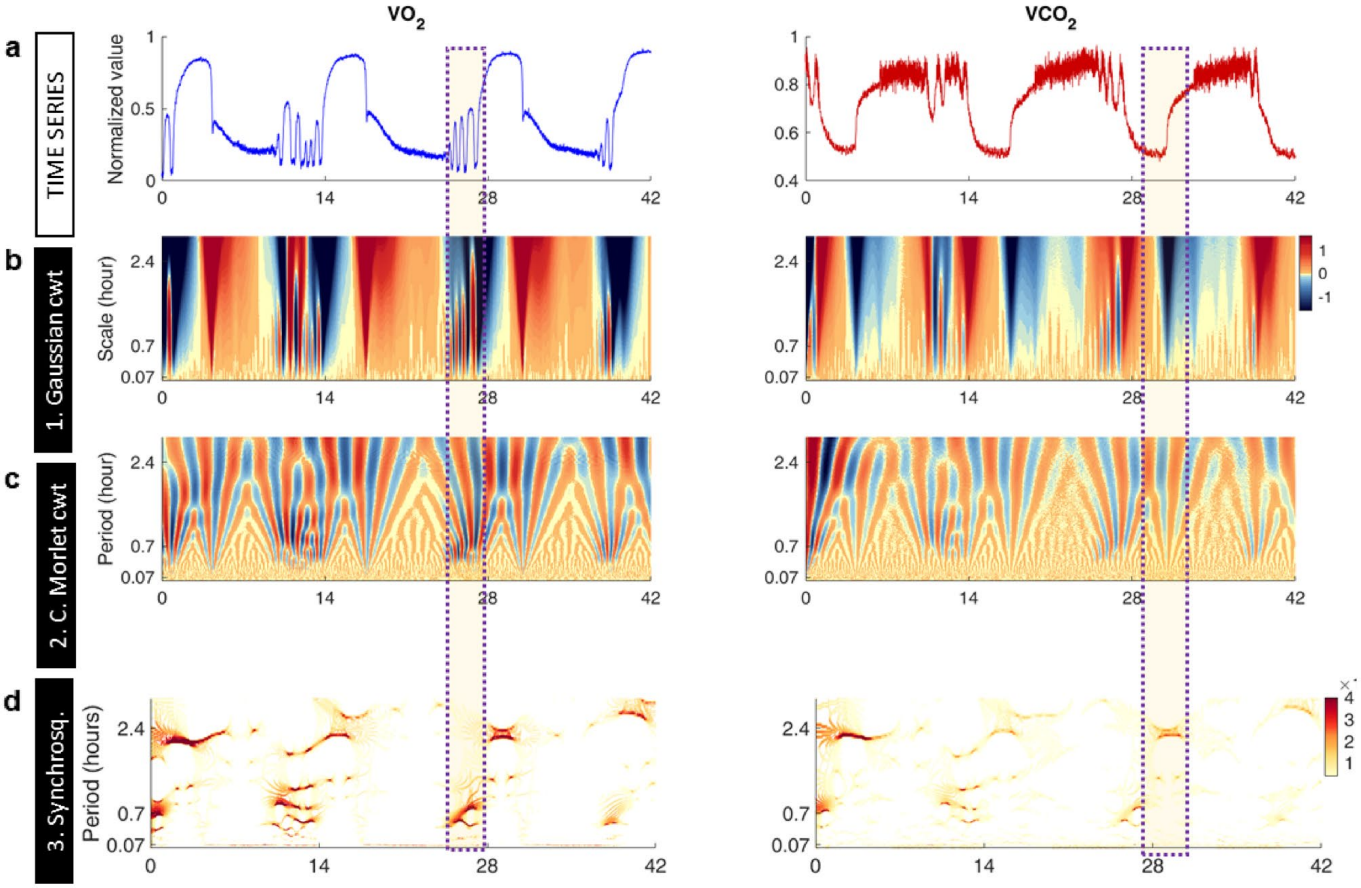
Detection and localization of higher frequency oscillations in O_2_ and CO_2_ signals obtained by MIMS from oscillating continuous cultures of *S. cerevisiae*. (**a**) Zoom in on the same time series shown in Fig. 1a of the relative MIMS signals of the m/z = 32 and 44 components vs time, corresponding to the O_2_ (blue) and CO_2_ (red) signals, respectively. Time is given in hours after start of the fermentor’s continuous operation. (**b**) Magnification of the same Gaussian cwt analysis shown in Fig. 1b. This wavelet highlights variability and transitions between states at a given time scale. Note the appearance of vertical lines around the 1 h time scale. (**c**) Magnification of the complex Morlet cwt analysis displayed in Fig. 1c (only the real part is shown). The bifurcation-like pattern marks the different periodic oscillations that compose the signal. (**d**) Magnification of the same wavelet synchrosqueezing analysis presented in Fig. 1d. Dark vertical localized bands indicate the estimated period of the signal at the time scales that appear in specific temporal windows. Note the thin red line at 4 min (0.07 h) period in the O_2_ synchrosqueezing coefficients (see zoom in Fig. S1). Color scale was adjusted to improve visualization.

Herein, we utilize GaMoSEC to detect and characterize periodicity in the time series of spontaneously self-synchronous yeast cell cultures as originated by Kuriyama’s group^57^. Under these conditions, yeast cultures can produce multiple frequencies when grown continuously under precisely controlled conditions^41^. Figures 1a, 2a, S1a and S2a show the time series of dissolved O_2_ and CO_2_ with different levels of magnification. Periods of 13 h, ~ 40 min and ~ 4 min have been previously reported utilizing power spectral and relative dispersional analyses^41,44^.

Figure 1 shows two different types of wavelets analyses, Gaussian (panel b) and complex Morlet (panel c) continuous wavelet transform (cwt). The colored rectangles highlight the correspondence between the time series (Fig. 1a, left (O_2_), and right (CO_2_) panels) and the wavelet analysis (Fig. 1b,c, left and right panels). In a first step of GaMoSEC, the Gaussian cwt shows the principal oscillation, in red orange, with a 13.6 h period in both signals, as well as apparent, faster-low amplitude oscillations (< 7 h; visualized in light brown pseudocolor (Fig. 1b, left and right panels).

In the second step of GaMoSEC, the complex Morlet cwt was applied. The real part of the coefficients of this wavelet (Fig. 1c) exhibits a remarkable branching pattern associated with the variety of slower and faster oscillations apparent in the time series. The value of the coefficients indicate regions associated with peaks (red) and valleys (blue) in the rhythm. For example, at the ~ 14 h period (see *y*-axis) positive coefficients (red peaks Fig. 1c left panel) are associated with low respiratory activity by yeast cells, i.e., high levels of dissolved O_2_ due to low uptake of the latter, while the low levels of dissolved CO_2_ are due to low release into the medium; the negative coefficients (blue, valleys, Fig. 1c left panel) denote high yeast respiration, i.e., low levels of dissolved O_2_ due to higher uptake of the latter, and high levels of CO_2_ due to its high release into the medium.

While the Gaussian cwt highlights variability and transitions between states at a given time scale, and the complex Morlet cwt the phase of the oscillation, the third step of GaMoSEC involves synchrosqueezing, which quantitatively describes the two main features characterizing an oscillatory signal, i.e., amplitude and period (= 1/frequency).

It also emphasizes their relationship, given that both changes in the period (y-axis) and the amplitude (pseudocolor scale) over time (x-axis) are visualized for the O2 (Fig. 1d, left panel) and CO2 (Fig. 1d, right panel) signals. Specifically, synchrosqueezing reveals which time points present in the time series attain a local maximum that determines a ‘ridge’ delineated by the main frequency of each rhythm within their own scale, ~ 14 h and ~ 7 h (Fig. 1d). Note the upward tendency of the ~ 14 h rhythm indicating a slight lengthening of the period over time.

The degree of variability of the ridge throughout the time series, indicating the stability and range of variation of the rhythm’s period over time, is also addressed by EWD, the fourth step of GaMoSEC (Fig. 1e). In this case, since the ~ 14 h period changes over time, EWD shows a mixture of horizontal lines at this time scale but not at ~ 7 h.

Wavelet coherence analysis, the fifth step of GaMoSEC, addresses the phase relationships between the O_2_ and CO_2_ time series from yeast cultures (Fig. 1f). Yellow in the color scale indicates strong coherence between signals, with a magnitude square coefficient > 0.7, while arrows denote phase relationships between the two signals at a given time scale. From ~ 14 h up to 2.4 h *strong coherence is observed.* In addition, arrows are all at around 180º indicating antiphase relationships between the O2 and CO2 signals (Fig. 1f). This is observable in the time series shown in Fig. 1g, at least for the slower time scales up but to a lesser extent beyond 2.4 h (Fig. 1h).

Figure 2 shows a magnification of the analytical approach shown in Fig. 1 focusing on faster temporal scales in order to visualize periodicities with periods from 0.07 h (~ 4 min) to 2.4 h (see also Fig. S1). The Gaussian wavelet shows the positioning of the faster, low amplitude, rhythm (dark red vertical regions) with respect to the slower, high amplitude, one (Fig. 2b). The complex Morlet cwt unveils the amplitude and frequency of the rhythms, depicting a branching pattern corresponding to 2.4 h and 0.7 h (~ 40 min) periods present in the time series (Fig. 2c). Note the branching from the ~ 7 h period depicted in Fig. 1c into the 2.4 h period shown in Fig. 2c. Synchrosqueezing further showed that the 2.4 h and 0.7 h periods are not constant, but rather intermittent, localized at specific time windows. Interestingly, the circahoralian ~ 0.7 h (~ 40 min) rhythm^46,47^ appears to be neither constant nor stable but present within localized and specific time frames (Fig. 2) with a slightly increasing period over time within a 30–40 min range, as previously described^44^. Interestingly, an even faster ~ 0.07 h rhythm is constant over time in the O_2_ time series but not in the CO_2_ time series (see higher magnifications in Figs. S1 and S2). In yeast, the CO_2_ dynamics observed at high-frequencies, display complex fluctuations without a well-defined period and intermittency, and correspond to time scales where predominant long-range correlations prevail, as shown in Fig. S3a and similar previous studies in rodents^59–61^ and quail^23,62^.

Together, the results show that spontaneously synchronized yeast cultures exhibit a dynamic pattern of ultradian metabolic rhythms with frequencies spanning from 14 h up to 0.07 h, as revealed by our 5-step wavelet approach, GaMoSEC. Importantly, the remarkable branching pattern noticeable in the Morlet cwt at distinct frequencies exhibits a self-similar, fractal nature, reminiscent of the organization of branching in trees, pulmonary and blood vessels^39,63^. Since the dynamic pattern of ultradian rhythms displayed by the self-synchronized yeast cultures exhibits a striking resemblance with those presented by quails’ locomotion^23^, we wondered about their generality as could be judged from the sharing of key organizational features.

### Shared dynamic pattern of circadian and ultradian rhythms by mammalian (mouse, rat) and avian (quail) organisms

To address the question of whether species with distinct evolutionary trajectories do exhibit similarity in the dynamic organization of their rhythms, we employed a similar analytical approach of time series as applied to yeast.

#### Time series of metabolism, movement, and food intake in isolated mice

We analyzed the time series data from Adamovich and colleagues^54^ corresponding to 3 months old C57BL/6 wild type fed ad libitum mice monitored in metabolic cages housed at 22 °C under 12 h light–dark regimen where the O_2_ consumption rate, CO_2_ release, spontaneous locomotor activity, and food intake were simultaneously measured^54^. ZT0 corresponds to the time lights were turned ON and ZT12 to the time lights were turned OFF in the animal facility^54^.

**Figure 3:**
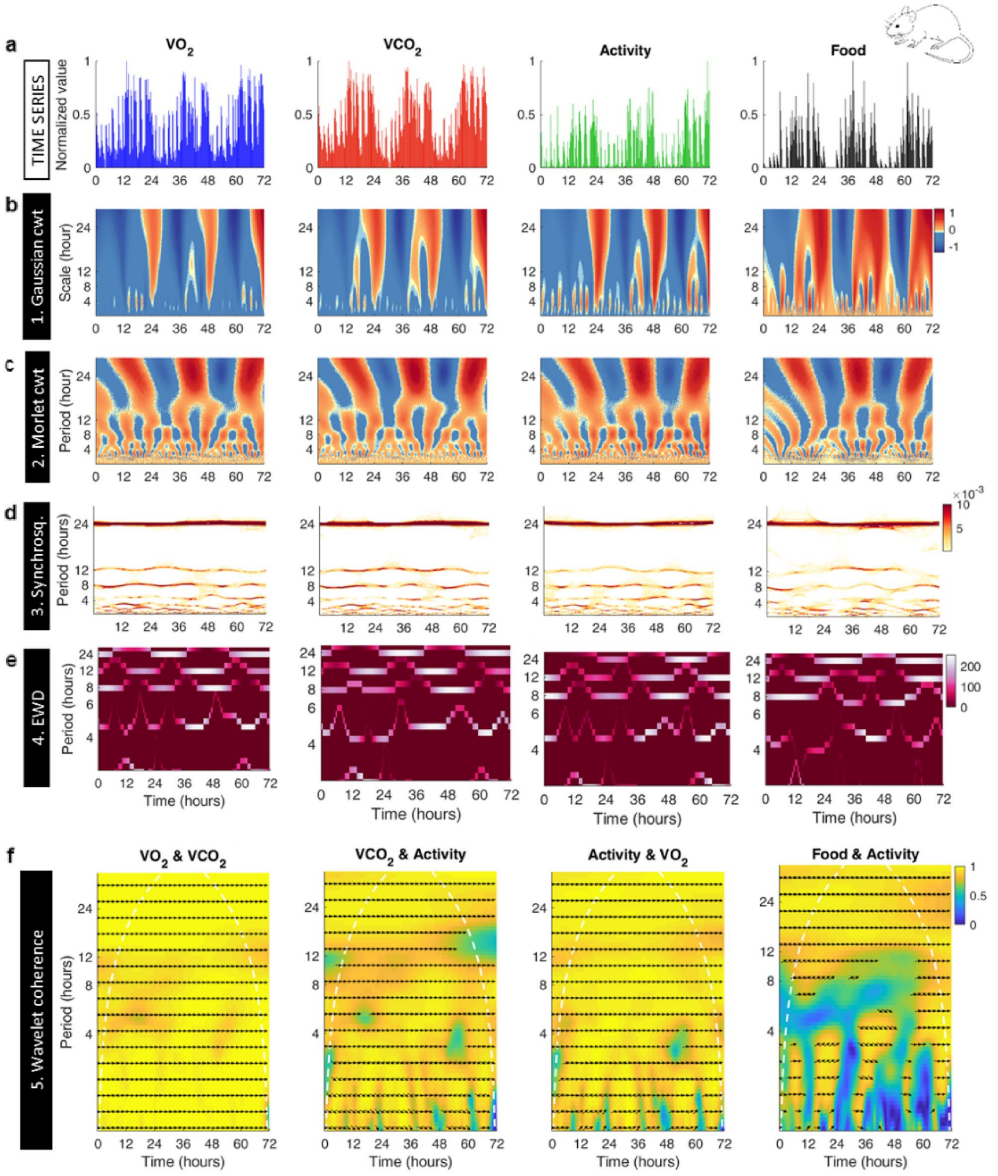
GaMoSEC analysis as applied to C57BL/6 wild type mice behavioral time series. (**a**) Wild-type female mice were housed under 12 h light–dark cycles. Metabolic cages were used to monitor oxygen consumption (VO_2_, blue), carbon dioxide (VCO_2_, red), spontaneous locomotor activity (green), and food intake (black). Data were recorded at 15 min intervals for 3 consecutive days. (**b**) Analysis of the time series shown in “a” with the Gaussian cwt. This wavelet highlights variability and transitions between states at a given time scale. Note that the principal circadian oscillation is observed in red over a broad range of scales. Fluctuations are visible for shorter time scales (< 12 h). (**c**) Analysis of the time series shown in “a” with the complex Morlet cwt (only the real part is shown). Note the bifurcation-like pattern highlighting the different periodic oscillations composing the signal. (**d**) Synchrosqueezing method applied to time series shown in “a”. Dark orange-red regions horizontal bands most noticeable around the 24 h, 12 h and 8 h time scales represent the circadian and two predominant ultradian rhythms, respectively. A band around 4.8 h is also observable. (**e**) Empirical Wavelet Decomposition applied to the time series shown in “a”, as well as horizontal bands most noticeable around the 24 h, 12 h and 8 h time scales represent the circadian and two predominant ultradian rhythm, respectively, consistent with Synchrosqueezing. A band around 4.8 h is also noticeable although its localization in frequency over time is not constant. (**f**) Wavelet coherence analysis between the four-time series. Color-scale represents the magnitude squared coherence between time series for a given time scale. Note the high positive values (yellow) observed at almost all scales. Arrows indicate phase relationships between signals at a given time scale. The 0° angle indicates that these series are completely in phase, as noticeable in the time series shown in panel a, for both slower and faster time scales. Mouse image: https://commons.wikimedia.org/wiki/File:Vector_diagram_of_laboratory_mouse_(black_and_white).svg.

Figure 3a displays a representative time series example of normalized O_2_ consumption and CO_2_ release rates along with activity and food consumption. The Gaussian wavelet shows cwt in the four-time series and the principal 24 h circadian rhythm in red orange, as well as apparent, faster-low amplitude oscillations (Fig. 3b). The real part of the Complex Morlet cwt also unveiled a branching pattern representing the variety of oscillations present in these time series (Fig. 3b). Note that pattern branching begins at the 24 h circadian rhythm as revealed by alternating peaks of night-time activity (red) and valleys of day-time inactivity (blue).

Synchrosqueezing (Fig. 3d) and EWD (Fig. 3e) confirmed the presence of 24 h, 12 h, and 8 h rhythms in this time series along with fluctuations at time scales below 6 h (see magnification in Fig. S4). The branching from the highest amplitude corresponding to the circadian rhythm was followed by the faster and lower amplitude ultradian rhythms in the rates of O_2_ consumption and CO_2_ emission (Fig. 3d,e, two left panels, respectively), activity and feeding (Fig. 3d,e, two right panels, respectively). The percentage of animals studied, in which these rhythms were detected along with the acrophase and power characterization of the 24 h, 12 h, and 8 h rhythms, are shown in Supplementary Fig. S5.

Wavelet coherence analysis showed that the O_2_ and CO_2_ time series from mice are largely in phase across time scales (Fig. 3f, left panel) as well as between both the dynamics of gases and activity (Fig. 3f, 2nd and 3rd panels). However, coherence between activity and feeding was consistent up to 12 h decreasing thereafter, especially at faster time scales (Fig. 3f, right panel).

Together, the ensemble of metabolic, spontaneous activity and feeding data in mice as well as rats (displayed in Supplementary Fig. S6) reveals a dynamic pattern of circadian and ultradian rhythms that shares a striking resemblance with those observed in yeast cultures. Like in yeast, the high-frequency complex fluctuations observed in mice and rats in the dynamics of gases and activity, characterized by the absence of a well-defined period and intermittency, correspond to long-range correlations as shown in Fig. S3b.

#### Behavioral time series in physically isolated quails

High-resolution time series of spontaneous locomotor activity (movement) along with feeding and drinking behavior from individually housed, Japanese quails (*Coturnix japonica*) were analyzed for 3 days sampled every 0.5 s, as previously described^23^.

**Figure 4:**
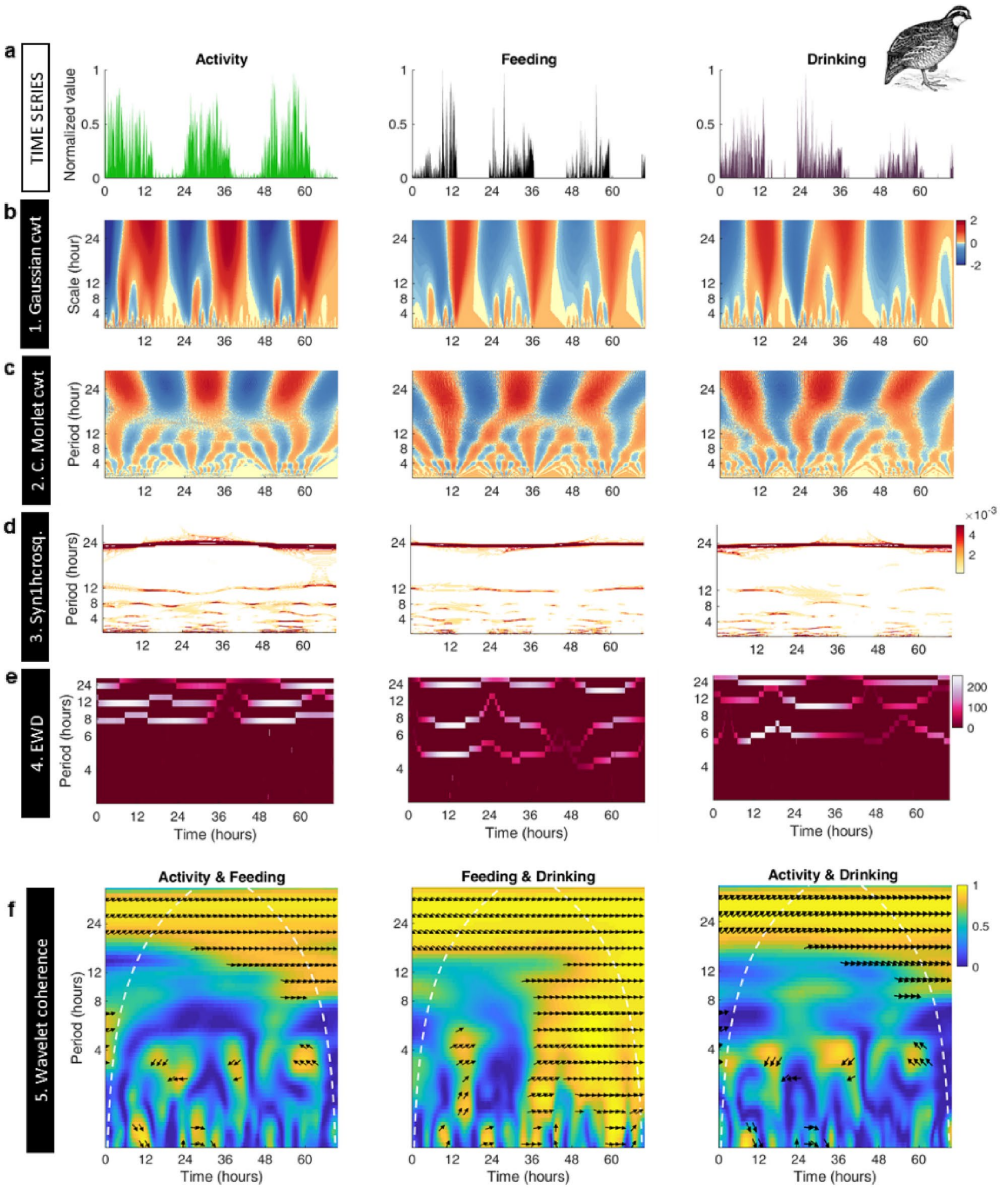
The 5-steps wavelet analysis of Japanese quail (*Coturnix japonica*) behavioral time series. (**a**) Spontaneous locomotor activity (green), food intake (black) and water drinking (purple) time series of an adult female Japanese quail in a home box environment. Data was obtained at a sampling rate of 0.5 s integrated in 6 min intervals. (**b**) Analysis of the time series shown in panel A with the Gaussian cwt. This wavelet highlights variability and transitions between states at a given time scale. Note that the principal circadian oscillation is observed in red over a broad range of scales. Fluctuations are visible for shorter time scales (< 12 h). (**c**) Analysis of the time series shown in panel (**a**) with the complex Morlet cwt (only the real part is shown). Note the bifurcation-like pattern corresponding to the different oscillations that compose the signal. (**d**) Wavelet synchrosqueezing method applied to time series shown in panel A. Dark orange-red regions horizontal bands most noticeable around the 24 h, 12 h and 8 h time scales represent the circadian and two predominant ultradian rhythms, respectively. A consistent and complete band over the 3-day experimental period for these ultradian rhythms could only be observed for locomotor activity. (**e**) Empirical Wavelet Decomposition applied to the time series shown in (**a**). Note the horizontal lines for the circadian rhythm are evident for the three-time series, while for  ultradian rhythms they are only well defined for locomotor activity consistent with synchrosqueezing. (**f**) Wavelet coherence analysis between the three-time series shown in (**a**). Color-scale represents the magnitude squared coherence between time series for a given time scale. Note the high positive values (yellow) are observed predominantly on the 24 h scales. Arrows indicate phase relationships between signals at a given time scale. The 0° angle indicates that these series are completely in phase. Image quail: https://commons.wikimedia.org/wiki/File:Quail_1_(PSF).png.

Figure 4a shows the normalized time series of activity (left panel), feeding (middle panel) and drinking (right panel). In the three variables measured, Gaussian wavelet analysis displays the circadian 24 h rhythm in red orange, as well as apparent, faster-low amplitude rhythms (Fig. 4b) whereas the complex Morlet shows the characteristic branching pattern of peaks (red) and valleys (blue) in correspondence with heightened activity and inactivity, respectively (Fig. 4c). Regarding locomotor activity, synchrosqueezing (Fig. 4d) and EWD (Fig. 4e) revealed the stability of the rhythms both in the circadian and ultradian domains, particularly noticeable at 12 h and 8 h, despite some variability between animals (see Fig. S7). Fluctuations were also detected in the faster time scales below 6 h (Fig. S8) and 3 h (Figs. S9, S10) where rhythms appeared localized in specific time frames and as expected, long-range correlations were also detected in the three time series of activity, feeding, and drinking (Fig. S3c)^23,62^. Finally, wavelet coherence analysis of the variables measured, presented a coordinated pattern only in the circadian but less so in the ultradian domain (Fig. 4f), especially between activity and feeding (Fig. 4f, left panel) or drinking (Fig. 4f, right panel) whereas, comparatively, the relationship between feeding and drinking exhibited higher coherence across temporal scales (Fig. 4f, middle panel).

Overall, the three species analyzed exhibited a striking similarity in the organization of the dynamic pattern of rhythms. In mammalian (mouse, rat) and avian (quail) species the circadian rhythm coexists with higher frequency-lower amplitude ultradian rhythms, irrespective of the variables measured. Moreover, the rhythms in metabolic behavior of yeast, and of activity and metabolism in both mice and rats, exhibited a high degree of coherence, stability and phase relationship over time.

### Origin of the dynamic branching pattern of circadian and ultradian rhythms

**Figure 5:**
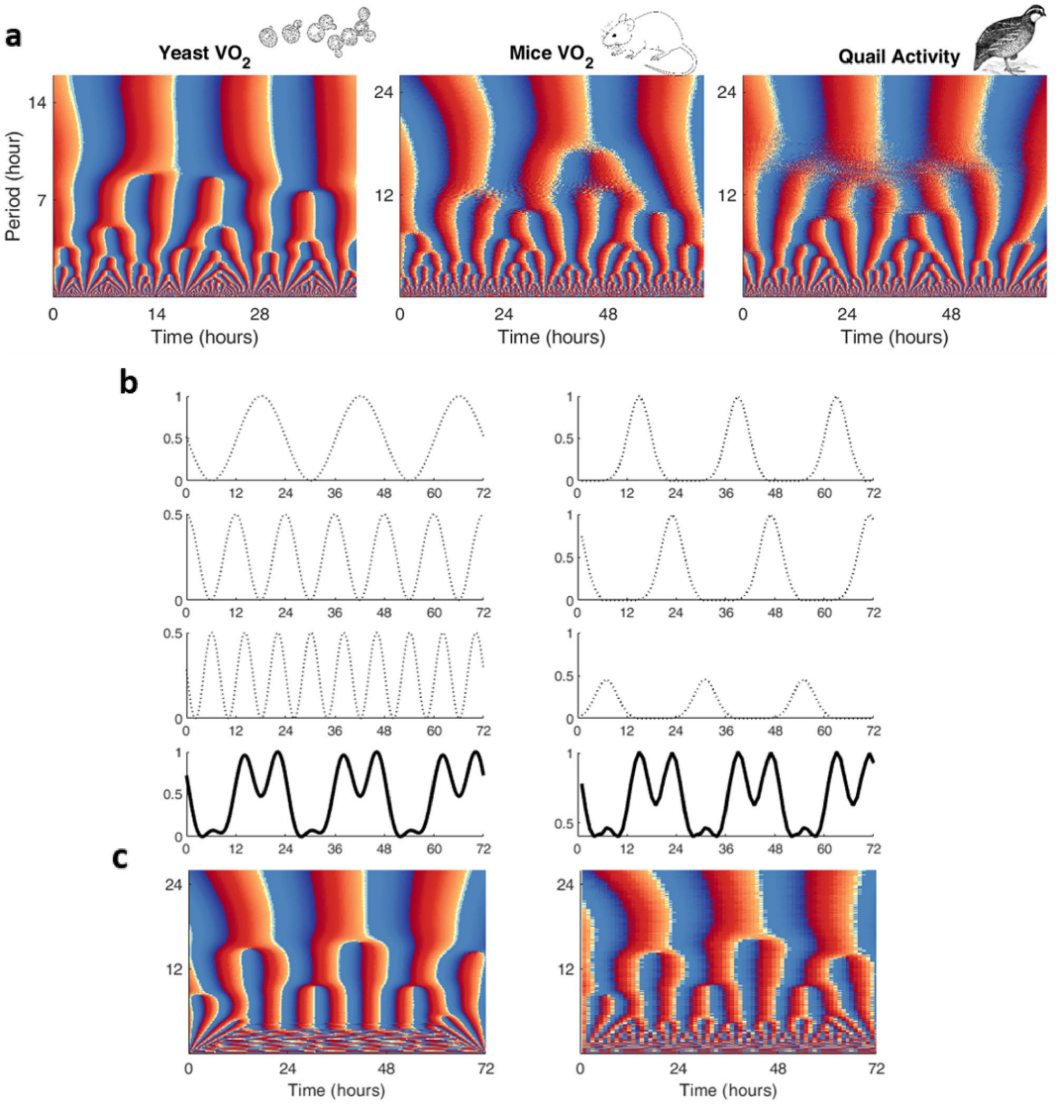
Resemblant dynamic pattern of circadian and ultradian rhythms in mice, quails and yeast can be understood by two distinct mechanisms. Visual comparison between time series analysis with the complex Morlet cwt (only phase angle is shown): (**a**) left panel, dissolved O_2_ obtained by MIMS from oscillating continuous cultures of *S. cerevisiae* (same as in Fig. 1a); middle panel, O_2_ consumption rate of wild-type female mice in metabolic cages (same as in Fig. 3a); and right panel, spontaneous locomotor activity of an adult female japanese quail in a home box environment (same as in Fig. 4a). (**b**) Two theoretical models that can give rise to the observed bifurcation patterns. On the left, three sinusoidal oscillations with different amplitudes and periods of 24 h, 12 h and 8 h were summed to create the synthetic time series shown in solid black lines on the bottom panel. On the right, three different trains of gaussian curves with peaks separated by 24 h periods are summed to create the synthetic time series (bottom panel). (**c**) Wavelet analysis of both synthetic time series. The same general bifurcation pattern is observed and of similar appearance as the time series from living organisms shown in panel “a”.

Next, we addressed the possible origin of the dynamic pattern of rhythms found, corresponding to a principal circadian oscillator apparently coexisting with ultradian rhythms which have frequencies that are multiples of the principal oscillator, as exemplified by the phase angle representations shown for VO_2_ in yeast, mice, quails in Fig. 5a, and for rats in Fig. S6c. The complex dynamics observed in yeast can be easily understood as a sum of sinusoidal oscillations (see Fig. S11, and^42^). However, for the time series from vertebrates, two possible hypothetical scenarios can underlie the dynamic pattern observed: (i) a sum of sinusoidal coexisting oscillations from circadian and ultradian origin (Fig. 5b, left column), or (ii) a sum of non-sinusoidal oscillations of circadian origin 24 h out of phase (Fig. 5b, right column). According to the former scenario, the subsequent ultradian rhythms (i.e., 12 h, 8 h) present characteristic phase and power relationships with regard to the circadian rhythm, whereas the second scenario postulates that only circadian rhythms happen to occur 24 h out of phase with each other. A similar branching pattern in the complex Morlet cwt arises in both scenarios (Fig. 5c), however, comparatively, the second is less parsimonious because it demands not only precise timing of the phase between peaks but also of their amplitudes.

**Figure 6:**
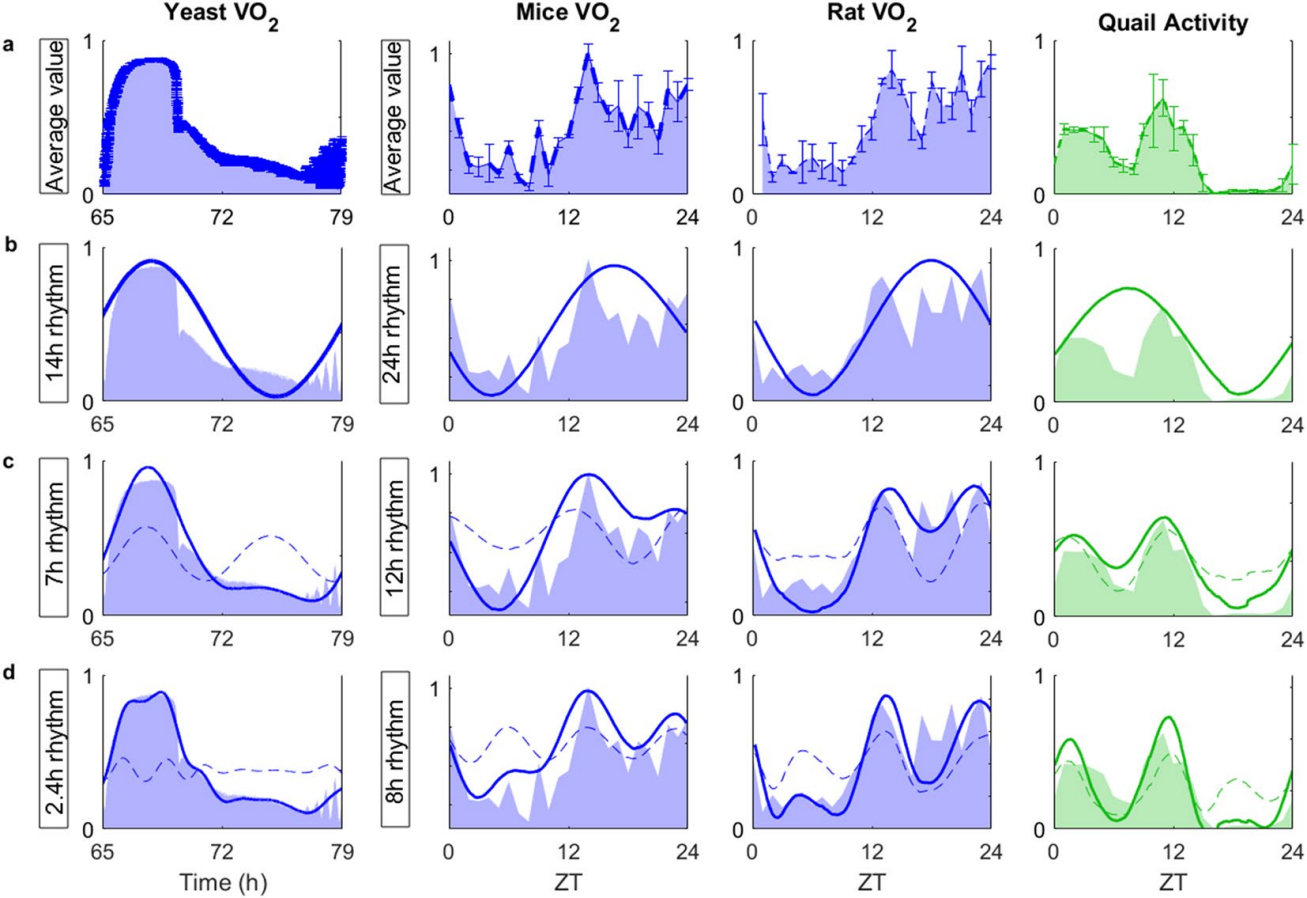
Contribution of circadian and ultradian rhythms to the temporal dynamics of O_2_ consumption (VO_2_) in yeast, mice and rat, along with locomotor activity in quail. Columns indicate animal model, and rows the following from top to bottom: (**a**) time series data is presented as the average of three periods (mean ± SEM) for a time interval of 0.2 min in yeast and 1 h in vertebrates. The colored area under the curve is maintained for comparison; (**b**) representation of the main (solid line) 14 h rhythm in yeast and 24 h circadian rhythm for vertebrates, according to Wavelet Synchrosqueezing (see Figs. 1, 3, S6, S4); (**c**, **d**) the 7 h and 2.4 h in yeast and 12 h and 8 h rhythms (dotted lines) determined with Wavelet Synchrosqueezing and shown consecutively to the circadian rhythm (solid line). UR, ultradian rhythm; ZT, zeitgeber time.

Further insight is provided in Fig. 6 where the analytical scheme of experimental data displayed in Figs. 1, 3, 4 and S6 are shown (time series shown as a filled area) superimposed with the extracted sinusoidal oscillations (bold colored lines). Importantly, the resultant pattern of 24 h, 12 h and 8 h is reproduced by wavelet analysis of the synthetic time series produced according to the first scenario (Fig. 5, compare panels a-left with panel c, left column). Moreover, the synthetic time series depicted in Fig. 5b (left column, bold trace) simulates the experimental time series of the rate of O_2_ consumption (VO_2_) in mice and rats (see Fig. 6, second and third columns, respectively, and Fig. S12 for mice where, in addition to VO_2_, CO_2_ release rate (VCO_2_), spontaneous locomotor activity, and food intake are plotted) and of quails’ activity (Fig. 6, fourth column). Yeast data, also included in Fig. 6 (left column), further supports the concept of rhythms coexistence at different temporal scales.

Together, the data presented suggest that the branching pattern deployed by circadian and ultradian rhythms, along with the phase correspondence between the different variables measured, agree with the idea that both time domains (circadian, ultradian) coexist.

## Discussion

The present work addresses the general question of contemporaneous rhythms in metabolism, movement, and feeding, spanning multiple time scales, and of its potentially uniform character in evolutionary distant organisms. The main finding shows that a unicellular eukaryote, such as yeast, as well as multicellular mammalian (mouse, rat) and avian (quail) organisms share key features of a dynamic coherent pattern of self-similar appearance in rhythms (Figs. 5a, S6c). In both an avian and mammalian species, this organization pattern temporally unfolds from the circadian domain, as can be judged by its branching appearance in the complex Morlet cwt given by splitting periods from 24 h into 12 h, 8 h, 4 h (Figs. 3, 4, S6c). In yeast, the splitting of the ~ 14 h cycle can reach down to 0.07 h (Figs. 1, 2). Besides experimental evidence, these findings are supported by synthetic time series corresponding to summation of coexisting oscillations from circadian and ultradian rhythm (Fig. 5a–c). Although most of previously published work focused on circadian rhythmicity, it is evident that the circadian domain of the temporal organization of animal behavior and metabolism is insufficient to understand the observed dynamic complexity (Fig. 6).

The unprecedented degree of detail attained in the description of the temporal scaling of the dynamic pattern described in the present work as applied to high resolution time series, could be achieved using our original five-step, wavelet-based, analytical approach^21^. The first step of GaMoSEC, consists of Gaussian cwt, critically important since it is based on a wavelet function that is *not in itself periodic*, thus fundamental for understanding data variability over different time scales while ruling out spurious harmonics^21^. Moreover, none of the time series analyzed in the present work shows evidence of waveforms that are discontinuous, spikes, square or strongly non-sinusoidal that would result in spurious harmonics. Hence, after the first step (Gaussian cwt), the subsequent analyses are based on the hypothesis that the data can be represented as a sum of sinusoids of different periods, which *do not involve harmonics* for representation. Complex Morlet cwt, synchrosqueezing, EWD and wavelet coherence analyses, unveil distinct key features of the rhythms present, such as amplitude, frequency, phase, power, temporal stability and coherence. Integrated, these methods allow not only simultaneous assessment of different possible rhythms as well as their temporal localization over a wide range of temporal scales (seconds to days), but also stability along with the phase correspondence between metabolism, locomotor and feeding activity of the animals (Figs. 3, 4, S4–S10), not possible with other methods traditionally utilized.

The emergent dynamic pattern of circadian and ultradian rhythms described in the present work, begets the fundamental question of coordination at the organismic level. Answering the coordination question entails on the one hand, knowing which rhythms emerge at the macroscopic scale and whether intermittent rhythms are happening sequentially or simultaneously. On the other hand, precisely, how, e.g., quails’ or mouse movements along with their metabolic and feeding behaviors are linked to SCN-mediated signaling of peripheral organs such as, e.g., cardiac and skeletal muscle, liver, pancreas, and brain.

Given the broad range of temporal scales covered by the dynamic pattern of rhythms, it is important to separate low-frequency high-amplitude from high-frequency low amplitude rhythms. In addition to the circadian rhythm, the first regimen comprises the ~ 12 h circatidal-like rhythm, not only documented in coastal organisms^64^ but also in the locomotor activity of quails^23^ and rats, as recently shown by Hasanpour and collaborators^65^, whom by comparing the circatidal-like rhythm with the artificial light/dark cycles (12L:12D) using phase-synchronicity analysis observed that the detected circatidal rhythm is unlikely to have been caused by the experimental setup. In addition, herein, we also show evidence of an 8 h rhythm in activity patterns. The analytical approach employed in the present work suggests that in mammalian and avian organisms there is a limit at ~ 4 h between low- and high-frequency rhythmic regimes. We propose that rhythms within the first regime correspond to dynamic events with *organismic* reach thus subjected to systemic modulation, likely of endocrine nature, like hormones and neurotransmitters via the circulation. The second regime includes behavioral temporal scales of ultradian rhythms that have been documented both in small mammals in the 2–4 h time scale (examples in Table S2), as well as avian species mostly at scales between 15 and 90 min (examples in Table S1). Results from previous studies point out the existence of identifiable control mechanisms for generation of circadian and ultradian rhythms^66^. Specifically, localized rhythms of high-frequency low-amplitude could emerge from the interaction with *local* biological subsystems within organs via paracrine modulation involving signaling and cell-to-cell communication through metabolites, second messengers, transcriptional factors. In this regime, where faster processes take place in the minutes to sec/msec scale, a panoply of feed-forward, -back, autocatalytic, allosteric, nonlinear mechanisms of control and regulation predominate.

We note that in the second, high-frequency, regime in yeast culture dynamics, intermittent periodicities happen within certain time frames, while nonexistent in others (see e.g., Figs. S1, S2). Unlike intermittent periodicities that in the yeast O_2_ signal occur sequentially over the 14 h time span, coexisting with a sustained high-frequency oscillation of 0.07 h (~ 4 min) period, the CO_2_ signal displays a wide variety of different fluctuations, likely presumably associated with fractal, scale-free dynamics^44^. In mammals and avian behavioral time series, intermittent periodicities without a well-defined period were also observed whereas sustained high-frequency oscillation over the entire 3-day period were not detected. This is consistent with previous ultradian rhythm studies where detectability and period can change throughout the day (Tables S1, S2). For example, locomotor ultradian rhythms of Siberian hamsters were more frequently detected during the daily dark phase^67^, and can even exhibit lower periods compared to the light phase^68^. In Siberian hamsters, depending upon the photoperiod and the light/dark phase, up to four significant distinct periods (between 0.1 and 7.9 h) could be detected^68,69^. Given the periods’ variability, Goh et al.^70^ proposed that rhythms in the range of 20 min–6 h should be more appropriately named Episodic Ultradian Events^70^. In agreement with our analytical approach, these authors also highlighted the importance of wavelet methods for understanding complex dynamics on faster time scales^70,71^.

Although research on biological dynamics has mainly focused on circadian rhythms, a large body of evidence shows that periodicities on fast and ultrafast time scales are not random and cannot be characterized by a single period. Instead, as shown in the present work (Fig. S3), long-range correlations associated with long-term memory, characterize these faster temporal scales^23,44,60,72,73^. Food restriction and injury in the suprachiasmatic nucleus (SCN) and/or dorsomedial hypothalamic (DMH) have been shown to differentially affect time scales >  ~ 4 h leading to random fluctuations that disrupt fractal activity patterns. Oppositely, these lesions induce only slightly more regular fluctuations at smaller time scales^60,61,74^. In this context, the SCN in the hypothalamus would coordinate temporal scales > ∼ 4 h whereas the second regime (i.e., < ∼ 4 h), would be more dependent on the organ and its function thus governed by local conditions. These reported results can be interpreted in the framework of our systemic (organism) and local (organ) proposal of rhythms coordination in the emergent pattern of rhythms described herein, according to which *systemic* regulatory mechanisms would set the stage for *local* events at the organ level. This hypothetical scheme implicitly suggests a *sequential* rather than a *simultaneous* mechanism of coordination (see^74^ for an alternative view).

## Concluding remarks

Together, the data ensemble shows that evolutionary distant species exhibit a strikingly similar pattern in the dynamic organization of rhythms in circadian and ultradian domains. The dynamic pattern’s resemblance, functional consistency, and broad occurrence as can be judged from mice, rats, quails and yeast analyzed, support its potentially universal character. Coherent behavior in metabolism concomitant with spontaneous or motivated movement (e.g., feeding, drinking) displayed phase relationships between the rates of O_2_ consumption and CO_2_ release such as in yeast and mice and, in the latter, in concordance with spontaneous and feeding behavior movement. Our highly sensitive methods were able to capture different degrees of coherence among rhythms such as in mice with respect to metabolic *vs.* activity or feeding, and in quails between movement *vs.* feeding or drinking. At the origin of the dynamic branching pattern of rhythms observed, our data support a scenario of coexistent and interdependent circadian and ultradian rhythms as shown by synthetic time series (Fig. 5) experimentally corroborated (Fig. 6). Hypothetically, the temporal unfolding and coordination of the branching pattern of frequencies (periods) in vertebrates can be modulated by rhythms of low-frequency high-amplitude at the organism level via endocrine processes with systemic reach, and locally, at the organ level, by high-frequency low-amplitude rhythms subjected to paracrine modulation.

## Methods

### Yeast, mice, rat and quail datasets

The yeast *S. cerevisiae* dataset^41^, as stated previously, has been analyzed with regard to rhythmicity using a different analytical approach^44^. In the present work, the same yeast time series were analyzed with the wavelet-based approach below described. Briefly, the yeast experimental time series correspond to an autonomously oscillating culture under constant environmental conditions (temperature, illumination, pH) and monitored by membrane-inlet mass spectrometry^75^. Data were collected every 12 s at m/z = 32, 34, 40 and 44 corresponding to oxygen, H_2_S, argon and carbon dioxide, where the m/z ratio, represents mass *m* divided by charge number of ions z. Argon m/z = 40 was used to correct for long-term drift in the instrument’s response as described previously^41^. The minutes temporal scale (0.07 h) present in self-synchronized cultures of yeast^41^, depicted herein in Figs. 1a and 2a, was confirmed by independent experiments in spontaneously synchronized oscillations in a contiguous layer of *S. cerevisiae* cells loaded with fluorescent probes, incubated at 30 °C with aeration of the perfusion buffer, and monitored by two-photon scanning laser fluorescence microscopy^44^. The yeast cells utilized in the two-photon experiments reiterate synchrony defined by cell sizing, flow cytometry and budding index of fixed aliquots of yeast cells removed at intervals from the long-term continuous cultures.

Yeasts were attached to a coverslip which had been coated with poly-L-lysine with unrestricted access to atmospheric oxygen on the stage of a Nikon E600FN upright microscope which was maintained at 30 °C^44,76^.

Both, mice (3 males, 3 females) and rats (8 males) datasets have been previously published and analyzed for circadian rhythms^54^ and were promptly supplied by the authors upon request. Individually housed mice and rats’ oxygen consumption and carbon dioxide release rates, spontaneous locomotor activity, and food consumption were simultaneously monitored using Phenomaster (TSE System) and Promethion (Sable Systems International) metabolic cages, respectively. Before each experiment, animals were adapted for several days (3–7 days acclimatization) in the metabolic cages enabling proper adjustment to the new housing conditions. Data was collected at 15 min intervals for TSE, and 1 min for Sable system. The light schedule in the metabolic cages was maintained as in the animals’ home cages using 12 light: 12 dark cycles. Fluorescent light of 100 lx intensity was applied for the metabolic cage recordings during the light phase^54^.

Quails were bred according to standard laboratory protocols^77^. The experimental protocol was approved by the Institutional Council for the Care of Laboratory Animals (CICUAL, Comité Institucional de Cuidado de Animales de Laboratorio) of the Instituto de Investigaciones Biológicas y Tecnológicas (IIByT, UNC-CONICET). Animal care and experimental treatments followed the Guide for the Care and Use of Laboratory Animals issued by the National Institute of Health (NIH Publications, Eighth Edition)^78^. They also followed local animal regulations including the Animal Protection law number 14346, National Administration of Drugs, Foods and Medical Devices (ANMAT) decree 6344/96, and the National Scientific and Technical Research Council (CONICET) resolution number 1047/2005. This study was carried out in compliance with the ARRIVE guidelines.

Quail datasets obtained from two independent experiments are publicly available, and results are shown in main text and Supplementary Figs. S5, S7–S10. Briefly, eight adult female quails were individually housed in 40 × 40  ×  40 cm (width × length × height, respectively) boxes with 3 solid white walls and one wire mesh wall that allowed visual contact with the female located in the contiguous box. Nylon monofilament line was extended over the top of the boxes with a 1 cm separation to prevent the birds from escaping without interfering with their visualization. A 14 light: 10 dark cycle was used. A video camera connected to a computer was suspended 1.5 m above the box. These cameras have built-in, infrared LED lighting, automatically switching to infrared recording after lights were turned off. Boxes had feeder, automatic nipple drinker, and floor with wood shavings. A period of at least 30 days acclimatization was taken before the actual recordings. The longer quail datasets used in Supplementary Fig. S10, were performed using a similar experimental setup but with the main exception that birds were visually isolated from each other, see complete description elsewhere^77^ and publicly available at FigShare (http://dx.doi.org/10.6084/m9.figshare.1424729) ANY-MAZE@ computer program was used to register locomotion at 0.5 s intervals (x_i_), and a customized application in Matlab was used to obtain feeding and drinking time series. The behavioral time series of each bird was obtained by assigning a number one (xi = 1) if during the interval the bird was performing the behavior, or a zero (xi = 0) if not^23^.

### Time series analysis with the 5-step wavelet approach GaMoSEC

Detection and characterization of oscillations were performed using a combination of 5 different wavelet decomposition techniques that simultaneously detrend and denoise the signal. Wavelets have the potential to describe the data without making any parametric assumptions about trends in the frequency or amplitude of the components signals and are resilient to noise (see review in^56^). Also, information regarding changes in temporal dynamics over the length of the experiment at different time scales is quantifiable. Hence, it is possible to detect the consolidation or disappearance of a given ultradian rhythm.  In wavelet analysis a specified function (i.e., wavelet) is compared to the signal at each time point. The resulting coefficients thus provide not only information regarding features of interest in the signal but also the temporal localization of that feature. Note that the wavelet used in the analysis depends on the features to be extracted, e.g., for a transition between states a Gaussian continuous wavelet transform (cwt) can be used, while for periodicity a complex Morlet cwt is appropriate. Consequently, wavelet analysis is not a single analysis but rather a family of analyses defined by the characteristics of the wavelet used in the transformation. Herein, the time series data were consecutively analyzed by the five types of transformations, coined GaMoSEC, an acronym describing the methodology used (i.e., Gaussian, Morlet, Synchrosqueezing, Empirical Wavelet Decomposition, and Wavelet Coherence). Code was written in Matlab and is publicly available at https://doi.org/10.684/m9.figshare.21545385.v1. Briefly, GaMoSEC comprises the following steps:

1. Visual inspection by continuous wavelet transform based on a real Gaussian mother wavelet in the Cartesian time scale plane. This wavelet transform highlights changes in the signal and singularities (i.e. spike-like or step-like changes) in the dynamics described by the time series, hence providing evidence of variability and fluctuations^21^.
2. Visual inspection by Continuous wavelet transform based on complex Morlet mother wavelet in the Polar time scale plane. This is a complex wavelet of periodic nature thus its transformation is also complex, providing 4 different plots corresponding to the real, imaginary, modulus and phase angle of the wavelet coefficients. This complex wavelet provides information about the presence of oscillatory behavior^21,23^ which can be used to estimate the acrophase^21^. Herein, only the real part of the transformation is shown.
3. Modal frequencies identification by synchrosqueezed wavelet transform is a linear time-scale analysis followed by a synchrosqueezing technique. This analysis provides highly localized frequency information, important for precise estimation of period and power of rhythms^21^.
4. Modal frequencies identification by Empirical Wavelet Decomposition, wavelet analysis in the Fourier domain followed by frequency segmentation to extract the modal components. This is an independent analysis that also can detect rhythms in time series, as well as changes in periodicity. The Matlab toolbox was made available by J. Gilles^79–81^.
5. Wavelet Coherence between pairs of different time series from the same individual and dataset was used to determine level of coherence as well as the phase relation between periodicities. For each animal, if evidence of periodicity was observed in the first four steps at a specific time scale (e.g., 24 or 12 h) and during a specific time period than the given rhythm, was considered to be detected under the specified experimental condition (Fig. 1). Percent of animals with the given rhythm was estimated.

The method utilized herein to determine scale-invariance and to evaluate the presence and extent of long-range autocorrelations in feed-intake and wheel running activity, was introduced by Peng et al.^82^ and is described in detail elsewhere^62^. Briefly, DFA estimates the self-similarity parameter that measures the autocorrelation structure of the time series. If α = 0.5, the series is uncorrelated (random) or has short-range correlations (i.e., the correlations decay exponentially), whereas 0.5 < α < 1 indicates long-range autocorrelation exist (correlation decays as a power-law), meaning that present depends on past behavior^83^. Also, α is inversely related to a typical fractal dimension, so in this case, the value increases with increasing regularity (or decreasing complexity) in the time series. This software is also available in the public domain (http://www.physionet.org/physiotools/dfa/). Herein, DFA calculations were performed with a customized script on MATLAB R2018a^84^.

### Materials and correspondence

Information and requests others than what are reported herein should be directed to Miguel A. Aon (miguel.aon@nih.gov) and Jackelyn M. Kembro (jkembro@unc.edu.ar).

## Supplementary Information

Supplementary Information.

## Data Availability

All data sets analyzed herein have been previously published^23,41,54^ with the exception of one of the quail datasets that is publicly available on Figshare, https://doi.org/10.6084/m9.figshare.21524481. Only animals from control groups, fed ad libitum, were analyzed herein. Data from yeast, rats and mice were made available upon request to the corresponding authors of the original manuscripts. Yeast data will be made available upon acceptance on FigShare. The longer quail datasets used in Supplementary Figure S10 are publicly available at FigShare (http://dx.doi.org/10.6084/m9.figshare.1424729).

## Code availability

Customized script in MATLAB will be made publicly available upon acceptance of publication in FigShare for GAMoSEC https://doi.org/10.6084/m9.figshare.21545385.v1. Customized script in MATLAB is currently publicly available on FigShare for Detrended Fluctuation Analysis (http://dx.doi.org/10.6084/m9.figshare.1514975). The Empirical Wavelet Transform Matlab toolbox was made available by J. Gilles, and is freely distributed on MATLAB Central File Exchange (https://www.mathworks.com/matlabcentral/fileexchange/42141-empirical-wavelet-transforms).
