# Supplementary material for: A dynamically coherent pattern of rhythms that matches between distant species across the evolutionary scale
Source: Sci Rep. 2023 Apr 1;13:5326. doi: 10.1038/s41598-023-32286-0 (PMC10067965; doi:10.1038/s41598-023-32286-0)
Supplement: Supplementary file 1 — Supplementary Information. [file 41598_2023_32286_MOESM1_ESM.pdf]

Supplementary Information

A dynamically coherent pattern of rhythms that matches between distant species across the evolutionary scale

Kembro, J.M[1-3#], Flesia, A.G.[4#], Nieto, P.S.[5#], Caliva, J.M.[1], Lloyd, D.[6], Cortassa, S.[7], Aon, M.A.[7,8 *]

[1] Consejo Nacional de Investigaciones Científicas y Técnicas (CONICET), Instituto de Investigaciones Biológicas y Tecnológicas (IIByT, CONICET-UNC), Córdoba, Argentina.

[2] Universidad Nacional de Córdoba, Facultad de Ciencias Exactas, Físicas y Naturales, Instituto de Ciencia y Tecnología de los Alimentos (ICTA), Córdoba, Argentina.

[3] Universidad Nacional de Córdoba, Facultad de Ciencias Exactas, Físicas y Naturales, Cátedra de Química Biológica, Córdoba, Argentina.

[4] Consejo Nacional de Investigaciones Científicas y Técnicas (CONICET), Centro de Investigación y Estudios de la Matemática (CIEM, CONICET-UNC), Universidad Nacional de Córdoba. Facultad de Matemática, Astronomía, Física y Computación Córdoba, Argentina

[5] Consejo Nacional de Investigaciones Científicas y Técnicas (CONICET), Instituto de Física Enrique Gaviola (IFEG, CONICET-UNC), Universidad Nacional de Córdoba. Facultad de Matemática, Astronomía, Física y Computación Córdoba, Argentina.

[6] Cardiff University, Schools of Biosciences and Engineering, Cardiff, Wales, United Kingdom

[7] Laboratory of Cardiovascular Science, National Institute on Aging, NIH, Baltimore, MD, USA

[8] Translational Gerontology Branch, National Institute on Aging, NIH, Baltimore, MD, USA

[#] The three authors contributed equally to this work.

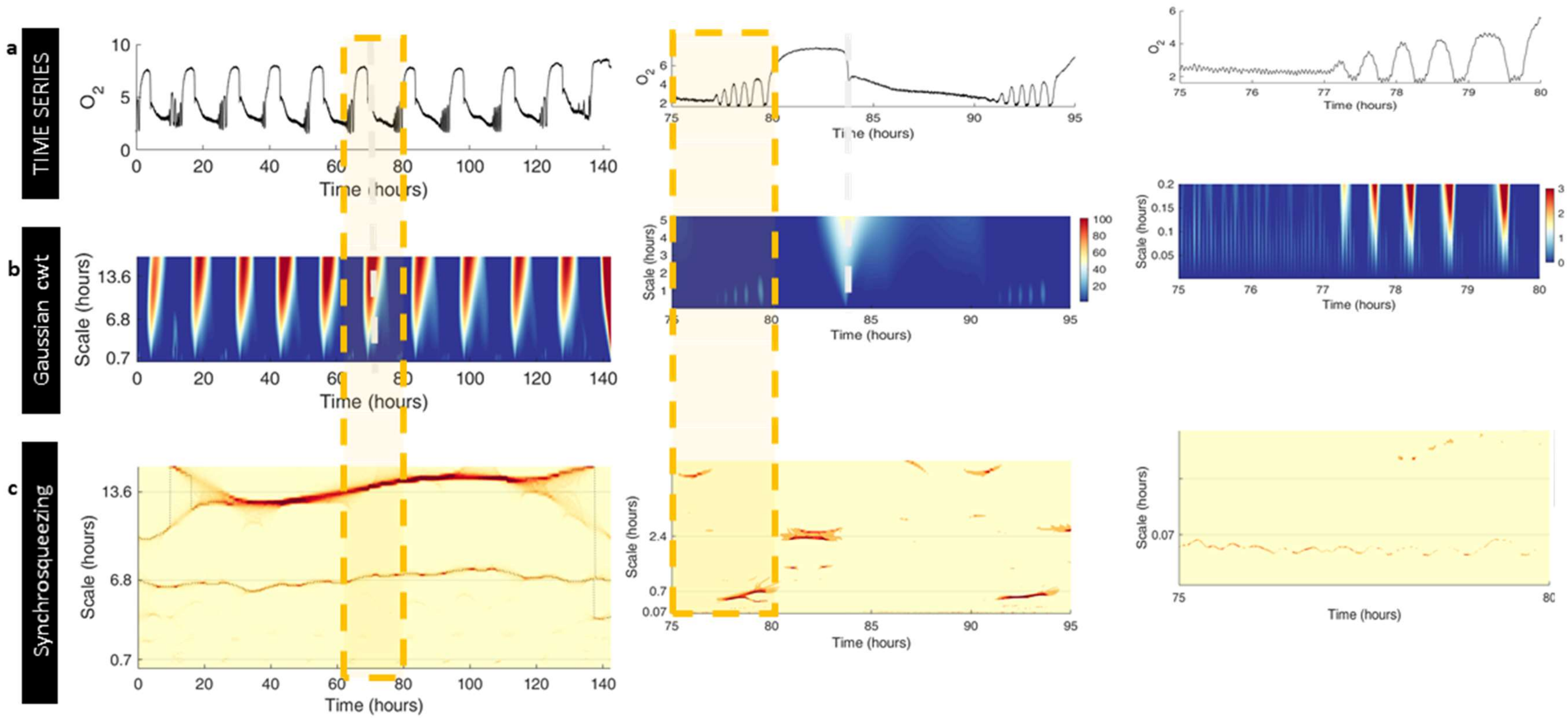

Supplementary Figure S1. Successive magnifications of the wavelet analysis of time series of O_2 and CO_2 signals obtained by MIMS from oscillating continuous cultures of *S. cerevisiae*

a) Relative MIMS signals of the m/z = 32 and 44 components vs time. Time is given in hours after the start of fermentor's continuous operation. Fermentors were run as described under Methods at a total volume of culture of 800 ml; medium flow rate, 1 ml/min, i.e., dilution rate, D= 0.0765 h^{-1}. The large-amplitude oscillation showed substantial cycle-to-cycle variability, with cycle times of 11.7 to 15.5 h, giving a mean of 13.661 h (SD, n = 8) [1].

b) Analysis of the time series shown in "a" with the Gaussian cwt. This wavelet highlights variability and transitions between states at a given time scale. Note that the principal oscillation is observed in red-orange over a broad range of scales. Fluctuations are visible for shorter time scales (<7h).

c) Analysis of the time series shown in "a" with the complex Morlet cwt. Given the complex nature of the wavelet, the real and imaginary part, modulus, and phase angle are shown. Note the bifurcation-like pattern in the real, imaginary and phase angle marking the different oscillations that compose the signal. The white and red horizontal bands in the modulus indicate periodicity at the corresponding time scale.

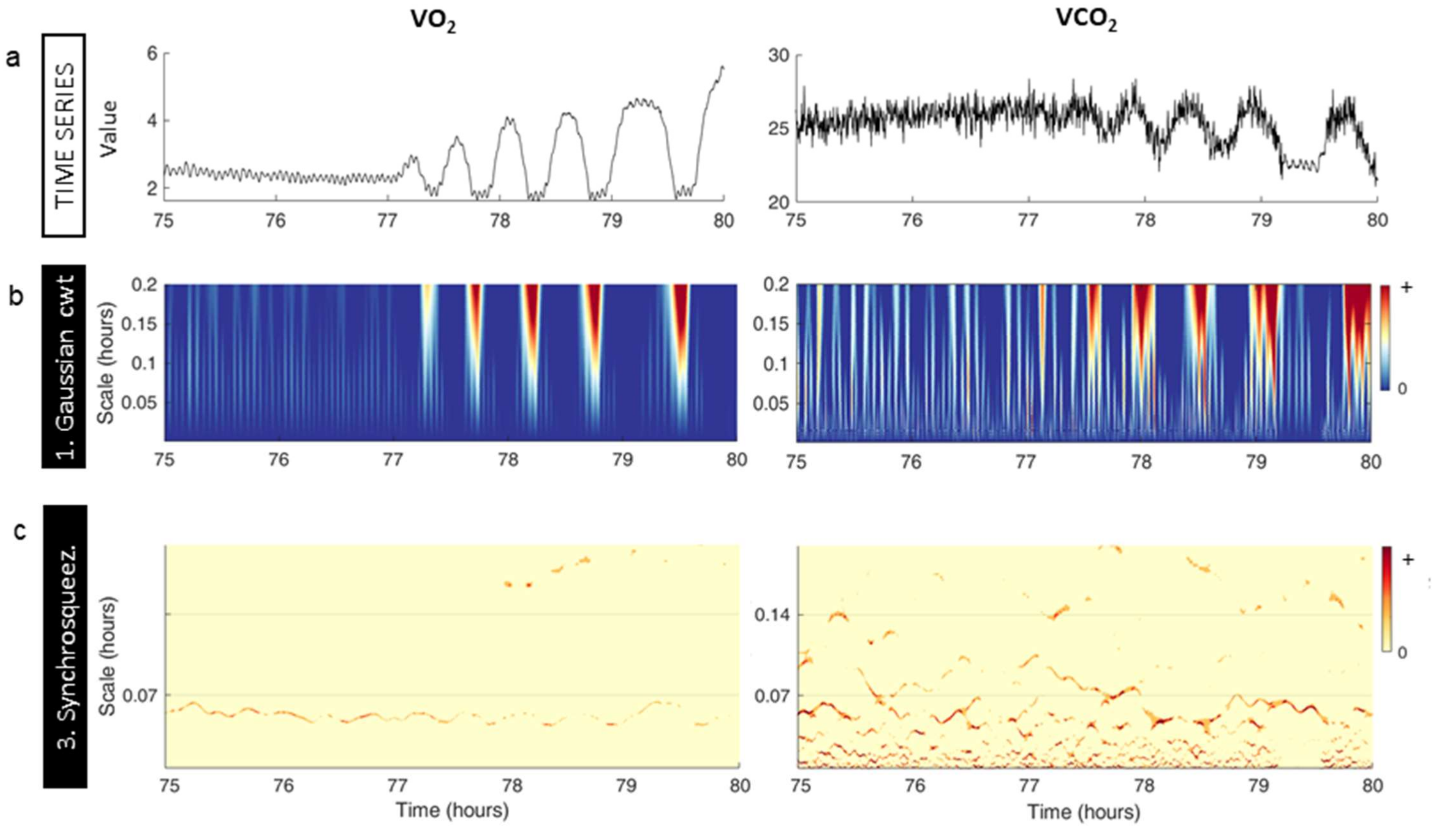

Supplementary Figure S2. High frequency oscillations are detected in O_2, but not CO_2 signals obtained by MIMS from oscillating continuous cultures of *S. Cerevisiae*

a) Magnification of the same time series shown in Fig 1a and 2a of the relative MIMS signals of the m/z = 32 and 44 components *vs* time. Time is given in hours after the start of fermentor continuous operation. This zoom in highlights the scales were the high frequency components are observable.

b) Magnification of the same Gaussian cwt analysis shown in Fig 1b and 2b. This wavelet highlights variability and transitions between states at this high frequency time scale. Vertical lines at these time scale represent fluctuations in the time series.

c) Magnification of the same wavelet synchrosqueezing analysis presented in Fig 1d and 2d. The red horizontal band close to the 0.07h time scale indicates periodicity in the O_2 signal, while not clearly apparent in the CO_2 signal. Color scales was adjusted to improve visualization.

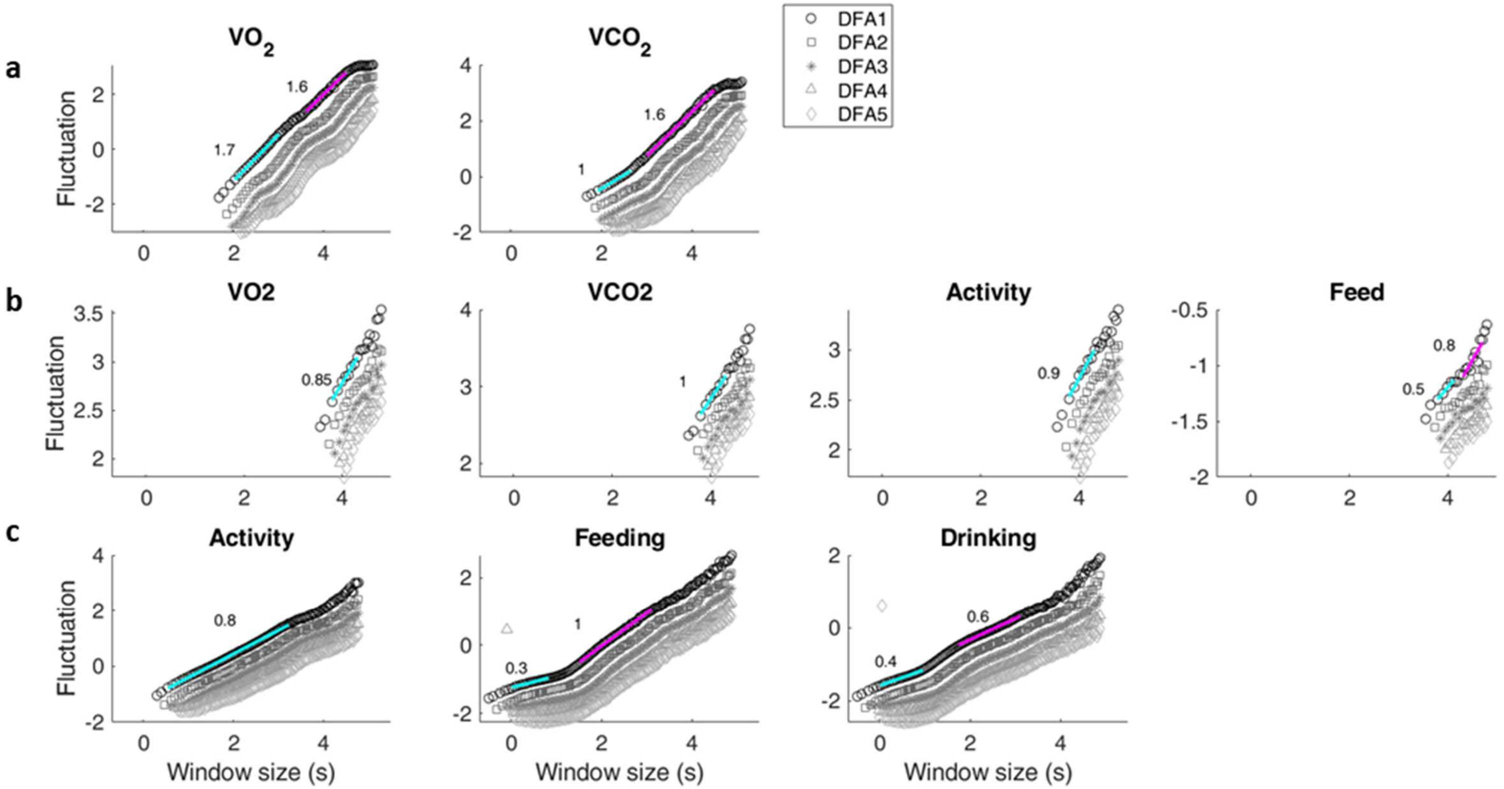

Supplementary Figure S3. Long-range correlations in time series from yeast, mice and quails.

Detrended Fluctuations Analysis (DFA) of time series of (a) oxygen and carbon dioxide concentrations obtained by MIMS from oscillating continuous cultures of *S. cerevisiae* (same as in Fig. 1a), (b) oxygen consumption rate (VO_2); carbon dioxide release rate (VCO_2) and spontaneous locomotor activity of wild-type female mice in metabolic cages (same as in Fig. 3a), and (c) Spontaneous locomotor activity, food intake and water drinking of an adult female Japanese quail in a home box environment (same as in Fig. 4a). DFA was performed using detrending orders 1-5 (DFA1-DFA5), as indicated with different symbols. Since all detrending orders presented similar plots, the first order (DFA1) was used for estimation. Colored lines indicate regions in which α-values (i.e., slope of the curve) was estimated using DFA1, and values are written nearside. Note the wide range of possible α- values estimated. However, in both mice and quail activity regions with long-range autocorrelation, with α- values between 0.5 and 1 are observable.

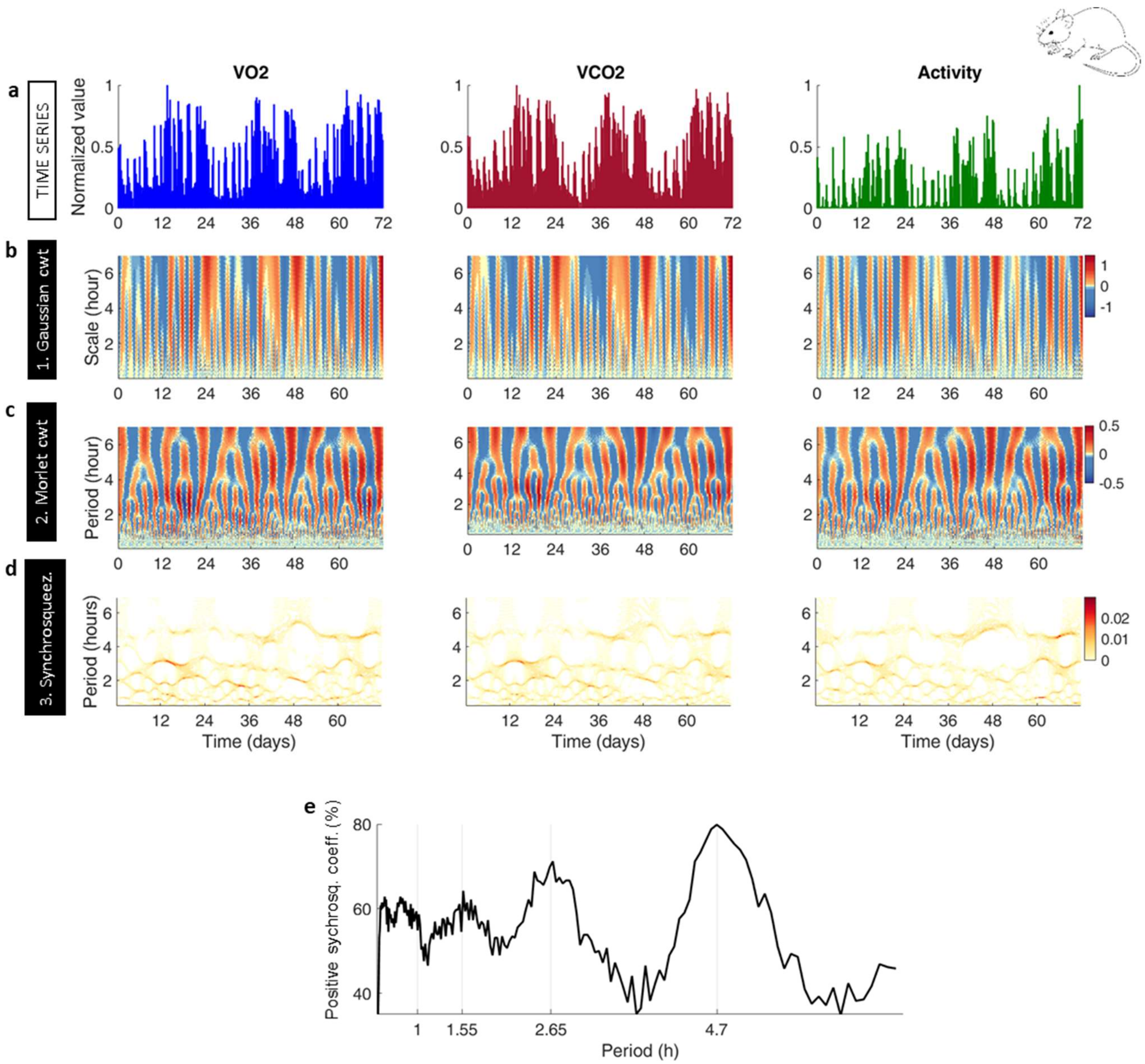

a
b
c
d
e
TIME SERIES
1. Gaussian cwt
2. Morlet cwt
3. Synchrosqueez.
VO2
VCO2
Activity
Normalized value
Scale (hour)
Period (hour)
Period (hours)
Time (days)
Positive sychrosq. coeff. (%)
Period (h)
1
1.55
2.65
4.7

Supplementary Figure S4. Detection of higher frequency oscillations with periods below 6h using GaMoSEC in C57/BL/6 wild type female mice time series of VO_2, VCO_2 and spontaneous locomotor activity.

a) For visualization purposes the same time series as in Figure 3 of main text are shown. Wild-type female mice were housed and monitored for oxygen consumption rate (VO_2, blue), carbon dioxide release rate (VCO_2, red) and spontaneous locomotor activity (green) as described in Fig. 3.

b) Analysis of the time series shown in “a” with the Gaussian cwt. This wavelet highlights variability and transitions between states at a given time scale. Fluctuations are visible for all time scales.

c) Analysis of the time series shown in “a” with the complex Morlet cwt (only the real part is shown). Note the bifurcation-like pattern denoting the different oscillations that compose the signal.

d) Wavelet synchrosqueezing method applied to time series shown in “a”. Dark orange-red regions horizontal bands are noticeable around period 2, 3 and 5, however the period is not well defined, changing over time.

e) Sum of wavelet synchrosqueezing coefficients shown in panel d that were estimated for each time scale. Peaks in the graph at around 2.65 and 4.7h are indicators of ultradian rhythmicity.

Image of mouse:
https://commons.wikimedia.org/wiki/File:Vector_diagram_of_laboratory_mouse_(black_and_white).svg

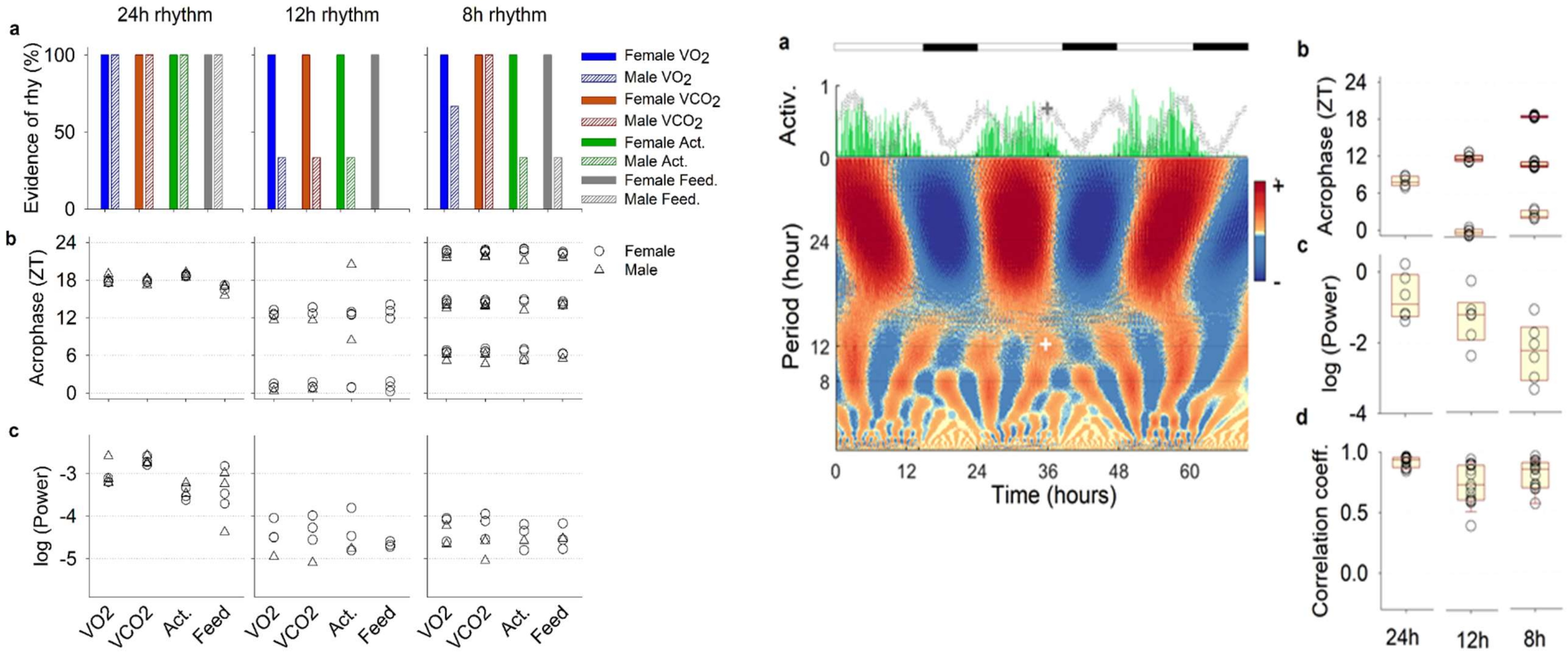

Supplementary Figure S5. Characterization of low frequency rhythms in individual mice behavioral variability (left column) and low frequency locomotor activity rhythms in Japanese quail (right column)

Left column: a) Percent of females (filled bars, n=3) and males (stripped bars, n=3) from the full time series dataset in which evidence of circadian, and 12h and 8h ultradian rhythms were detected using the 5-step wavelet approach GaMoSEC in oxygen consumption (VO_2, blue) carbon dioxide (VCO2, red), spontaneous locomotor activity (Act., green), and food intake (Feed, gray). b) Acrophase and c) Power estimation in time series in which rhythms were found, with female data represented with open circles and males with open triangles. The real part of the complex Morlet cwt was utilized to estimate the acrophase, i.e., the time at which the peak of a rhythm occurs, while the modulous squared value of the synchrosqueezing coefficient was used to estimate the power of the rhythm.

Right column: a) Example of spontaneous locomotor activity (green) time series were obtained from adult female Japanese quail in a home box environment under a 14L:10D cycle under standard environmental temperature conditions (24ºC, Control) and the corresponding real part of the complex Morlet cwt analysis. Superimposed on the time series in grey dotted lines the values of the real part of the complex Morlet cwt for the 12h period scale is shown, with the + denoting the maximum value associated with the acrophase of the 12h rhythm. b-d) Statistical analysis comparing treatment groups in regard with phase, power and correlation between animals estimated for the 24h, 12h and 8h rhythms in left, middle and right columns, respectively.

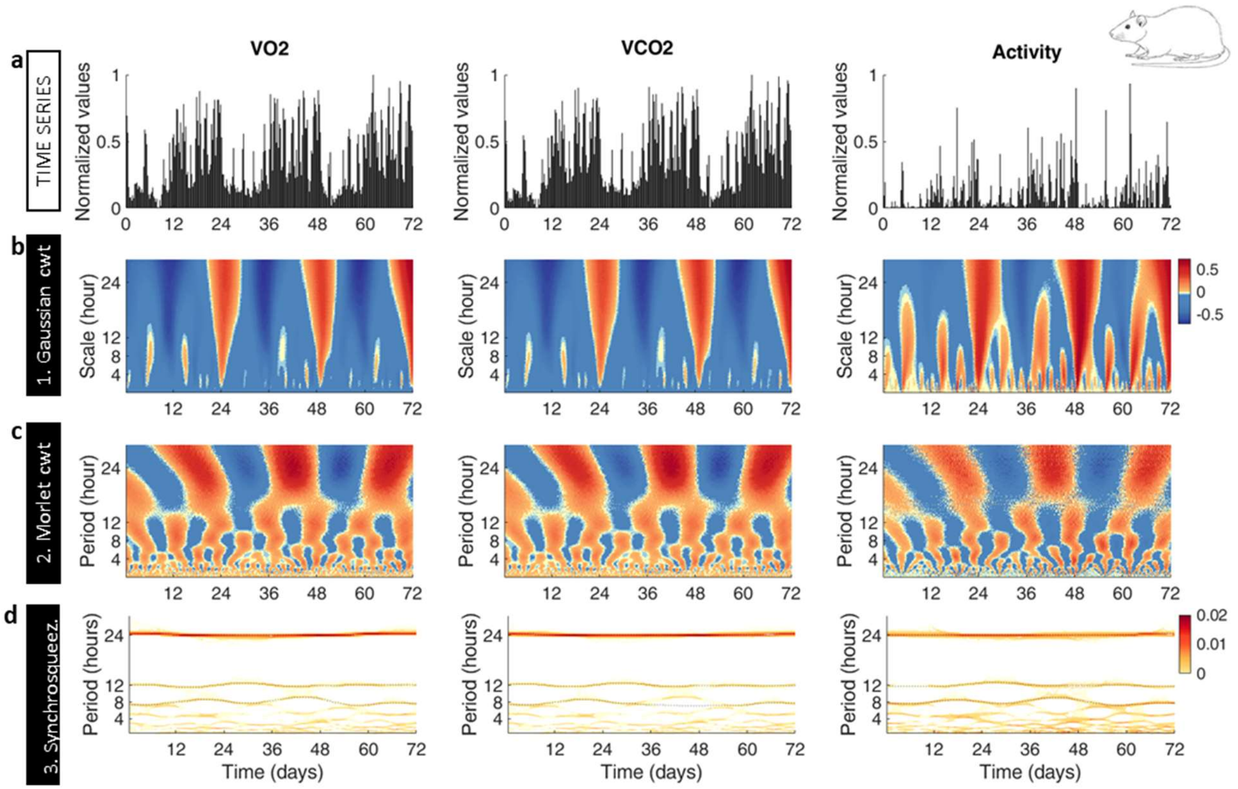

Supplementary Figure S6. Daily rhythms in oxygen consumption and carbon dioxide release in rats.
a) Rats were housed under 12 h Light-Dark cycles. Metabolic cages were used to monitor oxygen consumption rate (VO_2); carbon dioxide release rate (VCO_2); spontaneous locomotor activity. Data were monitored at 15 min intervals for oxygen, carbon dioxide and locomotor for 3 consecutive days.
b) Analysis of the time series shown in "a" with the Gaussian cwt. This wavelet highlights variability and transitions between states at a given time scale. Note that the principal circadian oscillation is observed in red over a broad range of scales. Fluctuations are visible for shorter time scales (<12h).
c) Analysis of the time series shown in "a" with the complex Morlet cwt (only the real part is shown). Note the bifurcation-like pattern denoting the different oscillations that compose the signal.
d) Wavelet synchrosqueezing method applied to time series shown in "a". Dark orange-red regions horizontal bands most noticeable around the 24, 12h and 8h time scale represents the circadian and the two predominant ultradian rhythm, respectively. A consistent complete band over the 3-day experimental period for these URs was only observed for locomotor activity.

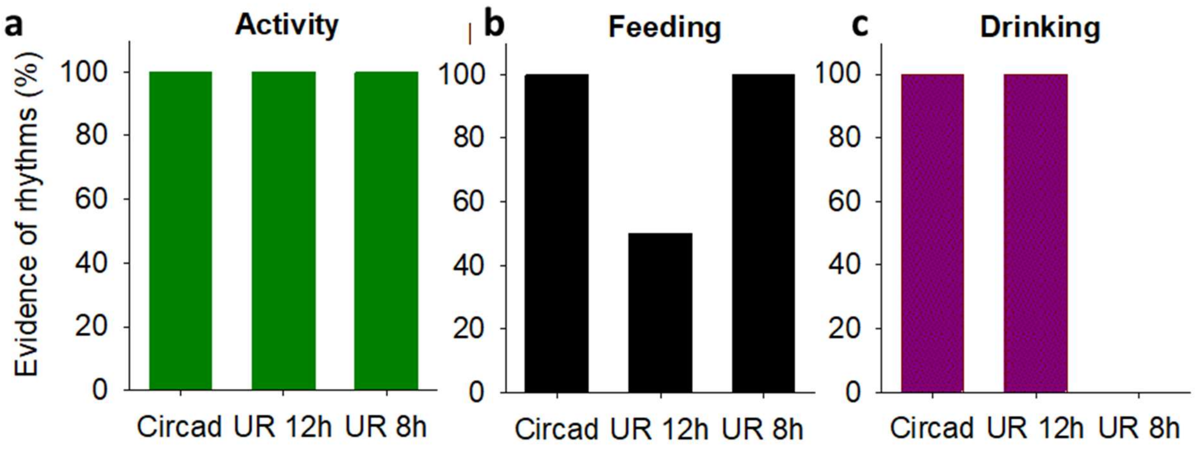

Supplementary Figure S7. Variability between individuals and behaviors in the detection of low frequency rhythms.

Percent of quail that presented evidence of circadian, and 12h and 8h ultradian rhythms (UR) detected using the 5-step wavelet approach GaMoSEC in a) Activity (n=8), b) Feeding (n=2) and c) Drinking (n=2) time series.

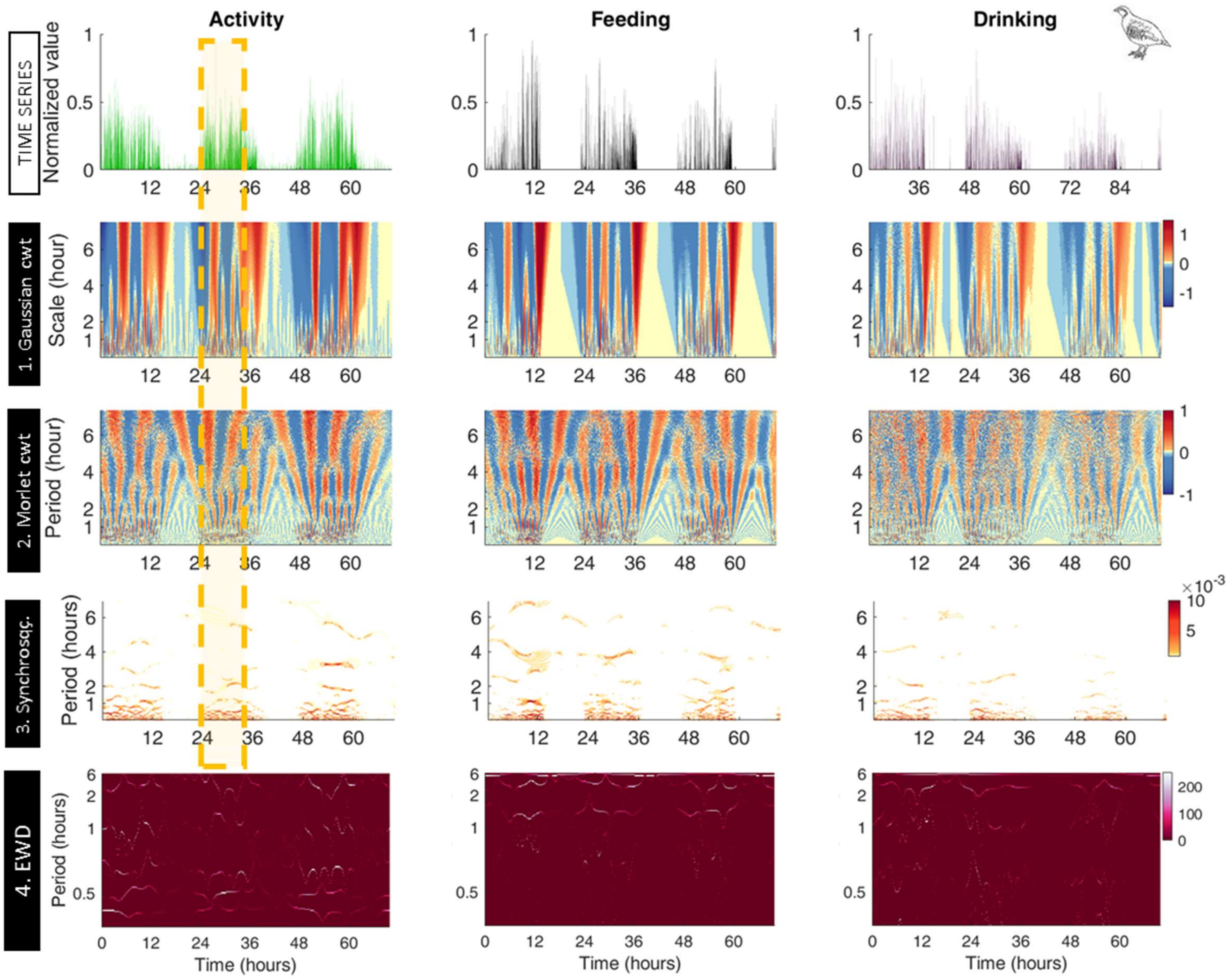

Activity
Feeding
Drinking
a
TIME SERIES
Normalized value
b
1. Gaussian cwt
Scale (hour)
c
2. Morlet cwt
Period (hour)
d
3. Synchrosqz.
Period (hours)
e
4. EWD
Period (hours)
Time (hours)

Supplementary Figure S8. Analysis of time scales below 6h in behavioral time series of Japanese quail (*Coturnix japonica*).
a) Same time series as shown in Figure 4 in main text. Spontaneous locomotor activity (green), food intake (black) and water drinking (purple) time series of an adult female Japanese quail in a home box environment. Data were obtained at a sampling rate of 0.5s, and binned into 1min intervals. Region amplified in Supplementary Figure S9 is marked with a yellow box.
b) Analysis of the time series shown in "a" with the Gaussian cwt. This wavelet highlights variability and transitions between states at a given time scale. The low prevalence of behavioral events during the nighttime in comparison to daytime is visible at these time scales, as shown by coefficients with a value of zero shown in yellow.
c) Analysis of the time series shown in "a" with the complex Morlet cwt (only the real part is shown). Note the bifurcation-like pattern denoting the different oscillations that compose the signal.
d) Wavelet synchrosqueezing method applied to time series shown in "a". A consistent complete band over the 3-day experimental period for these UR are not observed at these time scale, rather discontinuous dark orange-red regions horizontal bands are noticeable only during the daytime (see Fig. S9 for further magnification).
e) Empirical Wavelet Decomposition applied to the time series shown in "a". As with synchrosqueezing ("d"). Complete band over the 3-day experimental period for these ultradian rhythms are not observed at these time scales. Image quail: https:// commo ns. wikim edia. org/ wiki/ File: Quail_1_ (PSF). png

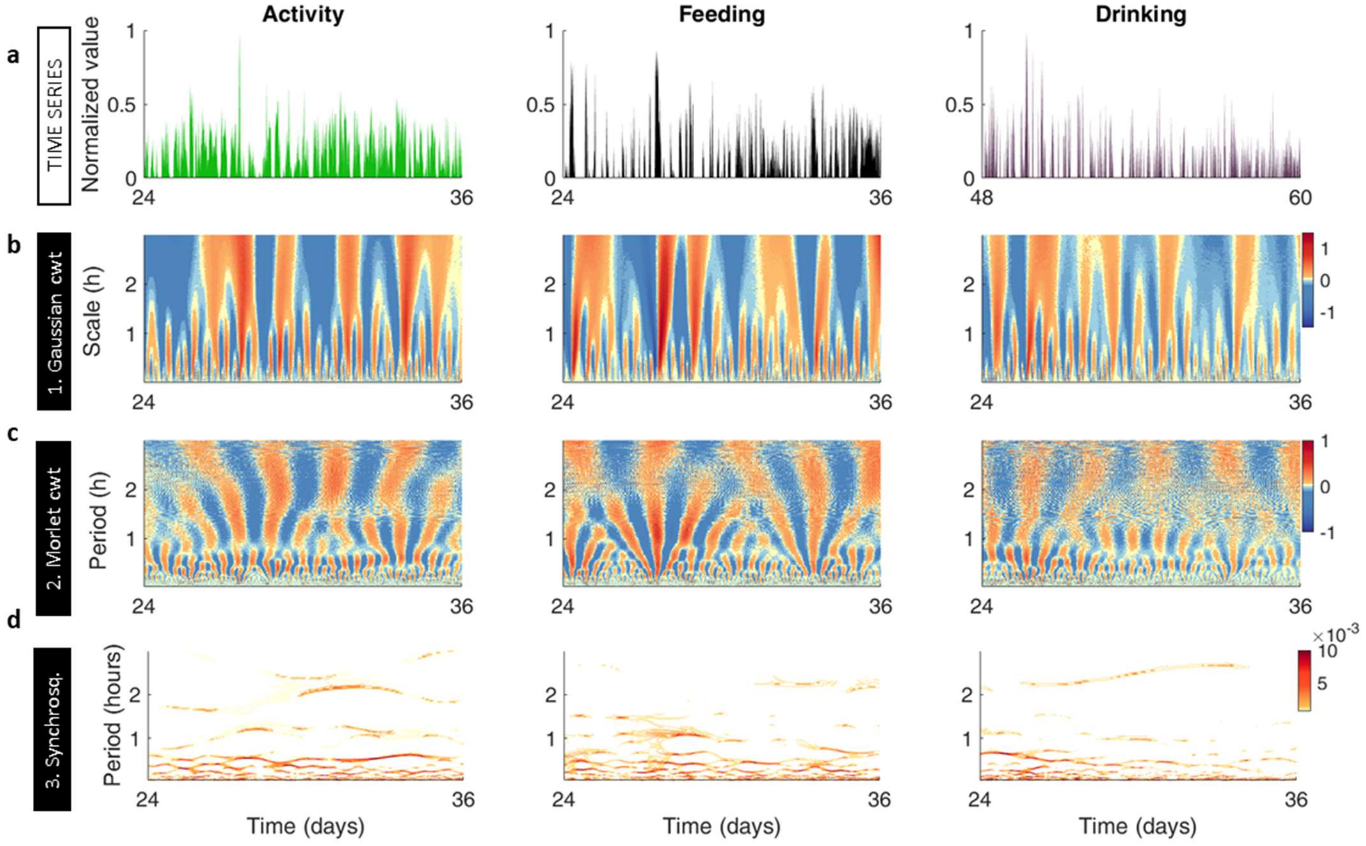

Supplementary Figure S9. Analysis of time scales below 3h in behavioral time series of Japanese quail, magnification of daytime activity.

a) The segment of the same time series shown in Fig. 4a and marked with a box in Figure S7a of spontaneous locomotor activity (green), food intake (black) and water drinking (purple) of an adult female Japanese quail in a home box environment is presented. Data were monitored at a sampling rate of 0.5s and binned into 1min intervals.

b) Magnification of the Gaussian cwt shown in Fig. 4b and S7b in order to visualize the variability and transitions between states at time scale bellow 3h. Note that the principal circadian oscillation is observed in red over a broad range of scales. Fluctuations are visible for these shorter time scales-

c) Magnification of the complex Morlet cwt analyses shown in Fig. 4c and S7c Note the bifurcation-like pattern continues at these time scales <3h.

d) Magnification of the wavelet synchrosqueezing analysis shown in Fig. 4 and S7d. Discontinuous horizontal bands are observed throughout the data. Period also shifts over time, for example in drinking a red horizontal band is observed, with the period slightly increasing over time.

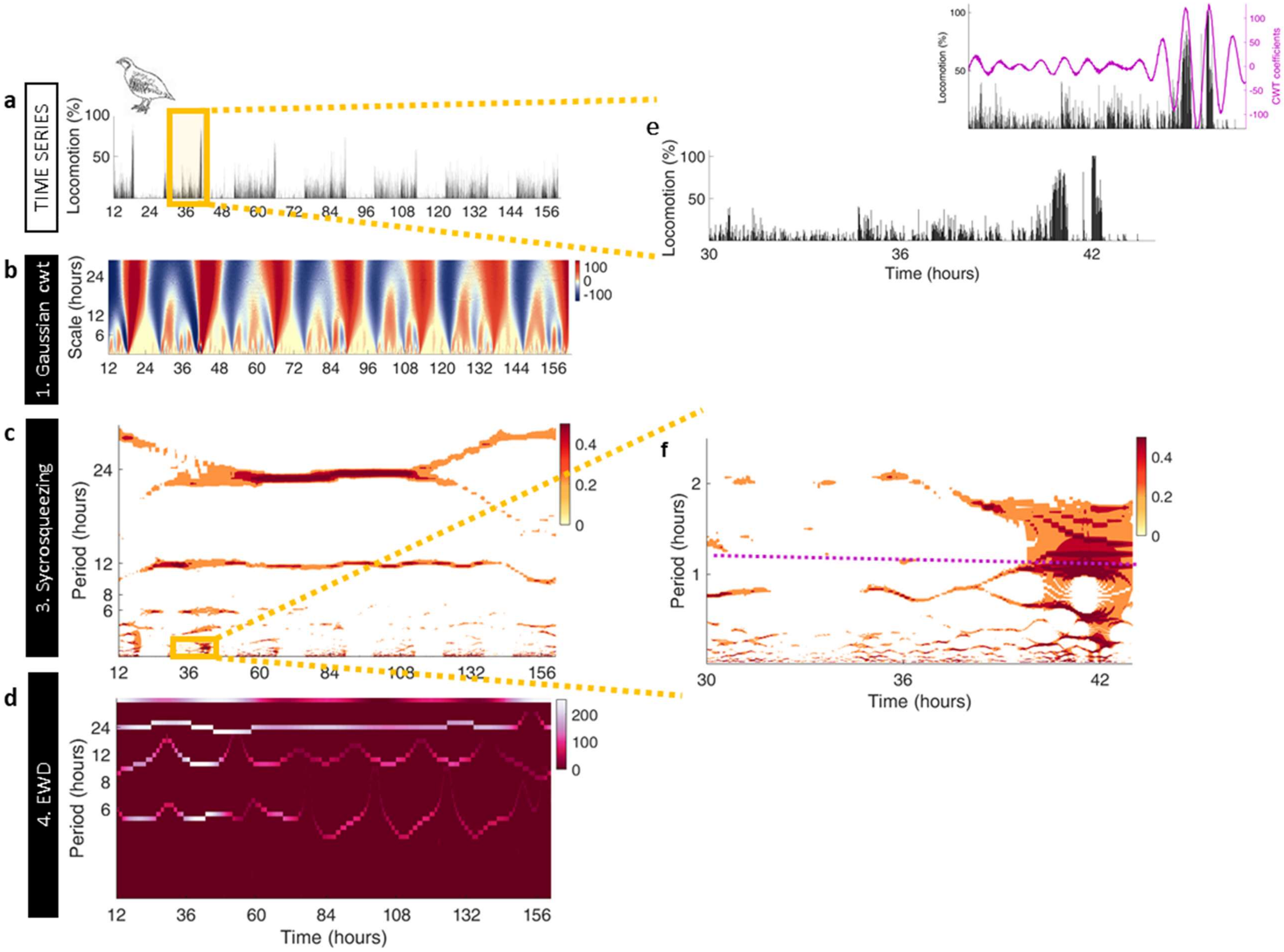
a
TIME SERIES
Locomotion (%)
Time (hours)
b
1. Gaussian cwt
Scale (hours)
c
3. Sycrosqueezing
Period (hours)
d
4. EWD
Period (hours)
Time (hours)
e
Locomotion (%)
CWT coefficients
Time (hours)
f
Period (hours)
Time (hours)

Supplementary Figure S10. Wavelet analysis applied to adult female quail locomotor time series

a) Locomotor time series of an adult female Japanese quail in a home box environment. Data corresponds to sampling rate of 0.5s and binned into 60s intervals. Example, quail 10 group11 (for details see ref.2 below).

b) Analysis of the time series shown in “a” with the Gaussian cwt. This wavelet highlights variability and transitions between states at a given time scale. Note that the principal circadian oscillation is observed over a broad range of scales. Fluctuations are visible for shorter time scales (<12h).

c) Wavelet synchrosqueezing method applied to time series shown in “a”. Dark vertical bands indicate the estimated period of the signal at the time scales around 24 and 12 hours.

d) Empirical Wavelet Decomposition applied to the time series shown in “a”. Horizontal white-pink bands indicate periodicities around the 24h, 12h and 6h time scales. As with the synchrosqueezing method, the 6h rhythm is only detectable during the first two days of testing.

e) Magnification of the time series shown in the yellow box in “a”.

f) Magnification of the wavelet synchrosqueezing method shown in the yellow box in “c”. Dark orange-red regions around the 1 h time scale denote the two peaks of activity that are separated by approximately 1.2h as indicated with purple lines in the inset of panel “e”.

Image quail: https:// commo ns. wikim edia. org/ wiki/ File: Quail_1_ (PSF). png

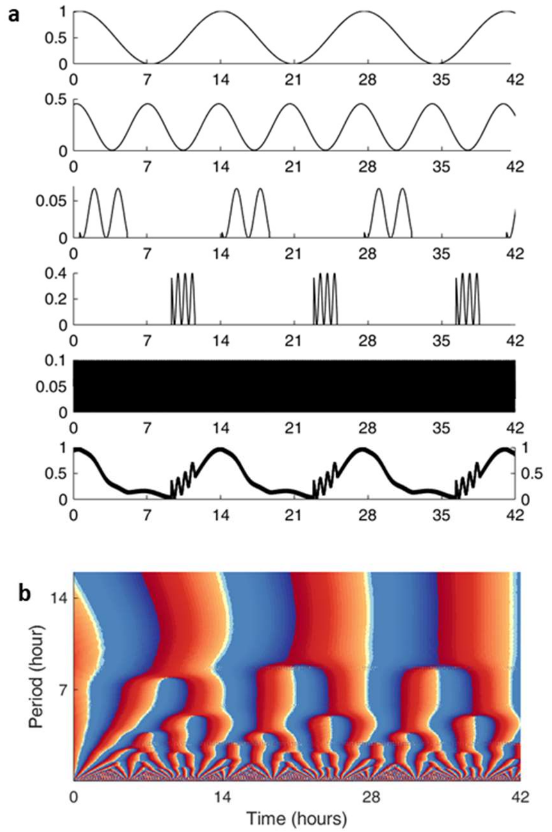

Supplementary Figure S11. A theoretical model that can give rise to the finer, higher resolution, details of the observed bifurcation pattern observed in yeast.

a) Five sinusoidal oscillations with different amplitudes and periods of 14, 7, 2.4, 0.7 and 0.07 h were summed to create the synthetic time series shown in solid black lines in the bottom panel (see similar experimental data in Fig. 2a, left panel).

b) Wavelet analysis of both synthetic time series. The same general bifurcation pattern is observed, similar to the analysis of time series from living organisms shown in Fig. 5a.

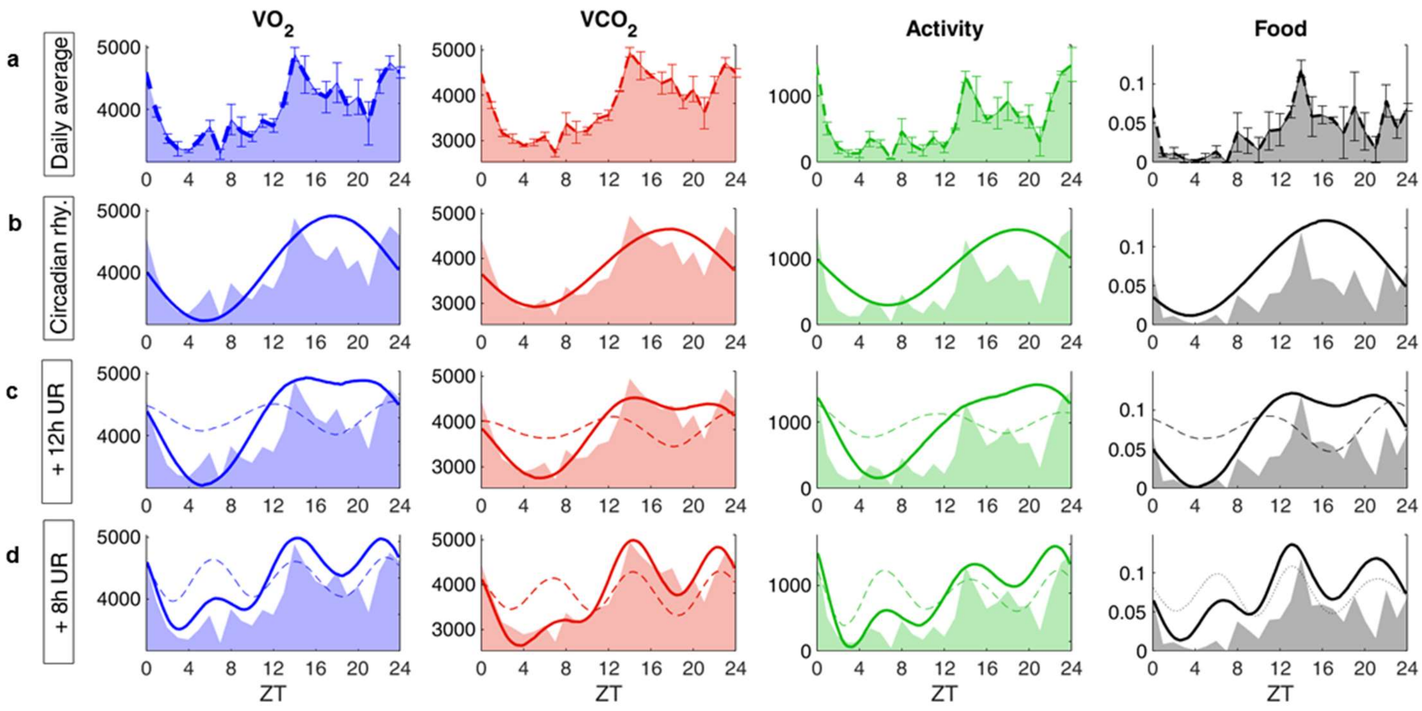

Supplementary Figure 12. Daily rhythms in O_2 consumption and CO_2 release in mice along with activity and food intake.
Wild-type female mice were housed under 12 h light-dark cycles. Metabolic cages were used for monitoring
a) The same mice time series shown in Fig. 4a, O_2 consumption rate (VO_2) (blue), CO_2 release rate (VCO_2) (red), spontaneous locomotor activity (green), and food intake (black). Data is presented as the days' average (mean ± SEM) during each 1h interval. The area under the curve was colored and maintained for comparison in panels b-d.
b) Representation of the circadian rhythms characterized with Wavelet Synchrosqueezing (see Fig. 3) is shown in the solid line. Note that peaks associated with onset and end of nighttime are not captured.
c,d) The 12h and 8h rhythms (dotted lines) characterized with Wavelet Synchrosqueezing were added consecutively to the circadian rhythm (solid line). ZT, zeitgeber time.

Table S1. Examples of reported ultradian rhythms (URs) in avian behavior

Period*	Specie	L:D	Age Group	Bahavior (sampling)	Method analysis	Variability	Social environ.	Observation	Ref.
**45 - 80 min**	Chicken (*Gallus gallus domesticus*) Ross 208, Ross 308, Labresse × ISA Brown, Labresse	LL	Newly hatched chick (first 3 days)	Activity in the groups (10 min)	Auto-correlation analysis	Detection of group with URs decreased from 17/20 to 7/20 over the 3 nights	Large groups (>90 chicks)	URs disappeared over time and little evidence of group rhythmicity remained by day 3.	3
**6 -40min**	Japanese quail (*Coturnix japonica*)	12.5L:11.5D	Chicks 4, 8, 12 and 15 days old	High/ low energy expenditure activities (3-min)	Autocorrelation, spectral analysis	Variability between groups in strength of rhythms	Groups of 12 chicks	Period lengthened as chicks grew older. After the 3rd week, the birds tended to become arrhythmic.	4
**14 -26min**	Japanese quail (*Coturnix japonica*)	12.5L:11.5D	Chicks (2 and 17 days old)	Activity (1-min) location every 2s was recorded; chicks could not be individualized.	Autocorrelation	UR in all groups	Groups of 4 chicks	~14 min when chicks were 2 days old to ~26.min when they were 16 days old	5
**45 min**	Japanese quail (*Coturnix japonica*)	10L:14D (also tested DD)	Juvenile	feeding activity (1min)	Autocorrelation	Detection of UR dependent on environmental conditions and genetics.	Individual		6,7
**15 min**	Japanese quail (*Coturnix japonica*)	14L:10D	Adults	Locomotor Activity (15 sec)	Wavelet analysis	Low interindividual variability	Groups of 1 male and 2 females	High level of synchronization between individuals of a group when an aggressive dominate was present	8
**4, 4.8, 6, 8, 12 h**	Japanese quail (*Coturnix japonica*)	14L:10D	Adult females	Locomotor activity (6 min, 1 min, 0.5 s)	Wavelet analysis	High inter-individual variability	Individual (visually isolated)		2

*Period of detected ultradian rhythms. Key to symbols: LL, under continuous light; DD, under continuous darkness; Ref., Reference.

Table S2. Examples of reported ultradian rhythms in mammalian behavior

Period*	Specie	L:D	Age	Bahavior (Sampling)	Method analysis	Variability	Social environ.	Observation	Ref.
2 – 3 h	Common vole (*Microtus arvalis*)	Different light regimens	Adult male and female	Feed-rest	X^2 periodogram		Visually isolated	No effects of deuterium oxide on URs nor direct synchronization by ultra-short light-dark cycles	9
3 h (2-5h)	Siberian hamsters (*Phodopus sungorus*)	Short day (SD), 9L:15D. Long day (LD) 15L:9D	Adult male	locomotor activity (6-min)	periodogram (LSP); cosinor periodogram (light and dark phases analyzed separately)	Not all exhibited UR and were evident in a greater proportion of SD than LD hamsters, and at night rather than day-time.	Individual		10-13
2 - 7h	Rat (SP) Winstar strain albino	12L:12D	Adult male	wakefulness, slow-wave sleep, paradoxical sleep (1h)	Power spectrum analysis	Large variability between animals	Group-housed until 2 days prior to test		14
1 - 2.5 h 12 h	Mouse (*Mus musculus*)	12L:12D	Adult male	Locomotor activity	Continuous wavelets transform	High inter-individual variability	Individual		15
4 h (3-5h)	Mouse (*Mus musculus*) 11 strains	12:12	Adult females	Locomotor (10-min)	autocorrelation analysis; MESA	65% presented UR, frequency depends on stain	Group-housed until test		16
6 – 15 h	Mouse (*Mus musculus*) WT, $mPer2^{Brdm1}$, Clock/Clock, and $Bmal1^{-/-}$	12L:12D	Adult males	Locomotor activity (1min)	Spectral analysis; continuous wavelet transform	High inter-individual variability	Individual	URs are relatively well maintained, only the Per2 mutant tend to show instability in UR.	17

*Period of detected ultradian rhythms. Key to symbols: LL, under continuous light; DD, under continuous darkness; Ref., Reference.

References

1 Aon, M. A. *et al.* The scale-free dynamics of eukaryotic cells. *Plos One* **3**, e3624, doi:10.1371/journal.pone.0003624 (2008).

2 Guzman, D. A. *et al.* The fractal organization of ultradian rhythms in avian behavior. *Sci Rep* **7**, 684, doi:10.1038/s41598-017-00743-2 (2017).

3 Nielsen, B. L., Erhard, H. W., Friggens, N. C. & McLeod, J. E. Ultradian activity rhythms in large groups of newly hatched chicks (Gallus gallus domesticus). *Behav Processes* **78**, 408-415 (2008).

4 Lumineau, S., Guyomarc'h, C. & Richard, J.-P. Ultradian Rhythm of Activity in Japanese Quail Groups under Semi-Natural Conditions during Ontogeny: Functional Aspects and Relation to Circadian Rhythm. *Biol Rhythm Res* **32**, 373–388 (2001).

5 Lumineau, S., Guyomarc'h, C. & Richard, J. P. Ontogeny of the ultradian rhythm of activity in Japanese quail. *Chronobiol Int* **17**, 767-776 (2000).

6 Formanek, L., Richard-Yris, M. A., Houdelier, C. & Lumineau, S. Epigenetic maternal effects on endogenous rhythms in precocial birds. *Chronobiol Int* **26**, 396-414, doi:10.1080/07420520902892433 (2009).

7 Formanek, L. & Lumineau, S. Individual behavioural rhythmicity is linked to social motivation in Japanese quail. *Appl Anim Behav Sci* **121**, 126-133, doi:10.1016/j.applanim.2009.09.004 (2009).

8 Alcala, R. S., Caliva, J. M., Flesia, A. G., Marin, R. H. & Kembro, J. M. Aggressive dominance can decrease behavioral complexity on subordinates through synchronization of locomotor activities. *Commun Biol* **2**, 467, doi:10.1038/s42003-019-0710-1 (2019).

9 Gerkema, M. P., Daan, S., Wilbrink, M., Hop, M. W. & van der Leest, F. Phase control of ultradian feeding rhythms in the common vole (Microtus arvalis): the roles of light and the circadian system. *J Biol Rhythms* **8**, 151-171, doi:10.1177/074873049300800205 (1993).

10 Prendergast, B. J., Cable, E. J., Cisse, Y. M., Stevenson, T. J. & Zucker, I. Pineal and gonadal influences on ultradian locomotor rhythms of male Siberian hamsters. *Horm Behav* **63**, 54-64, doi:10.1016/j.yhbeh.2012.11.001 (2013).

11 Prendergast, B. J., Cisse, Y. M., Cable, E. J. & Zucker, I. Dissociation of ultradian and circadian phenotypes in female and male Siberian hamsters. *J Biol Rhythms* **27**, 287-298, doi:10.1177/0748730412448618 (2012).

12 Prendergast, B. J., Stevenson, T. J. & Zucker, I. Sex differences in Siberian hamster ultradian locomotor rhythms. *Physiol Behav* **110-111**, 206-212, doi:10.1016/j.physbeh.2013.01.008 (2013).

13 Prendergast, B. J. & Zucker, I. Photoperiodic influences on ultradian rhythms of male Siberian hamsters. *Plos One* **7**, e41723, doi:10.1371/journal.pone.0041723 (2012).

14 Ibuka, N., Inouye, S.-i. T. & Kawamura, H. Analysis of sleep-wakefulness rhythms in male rats after suprachiasmatic nucleus lesions and ocular enucleation. *Brain Res* **122**, 11, doi:10.1016/0006-8993(77)90660-6 (1977).

15 Poon, A. M. S. *et al.* Effect of cage size on ultradian locomotor rhythms of laboratory mice. *Physiol Behav* **62**, 1253-1258 (1997).

16 Dowse, H., Umemori, J. & Koide, T. Ultradian components in the locomotor activity rhythms of the genetically normal mouse, Mus musculus. *J Exp Biol* **213**, 1788-1795, doi:10.1242/jeb.038877 (2010).

17 Nakamura, T., Takumi, T., Takano, A., Hatanaka, F. & Yamamoto, Y. Characterization and modeling of intermittent locomotor dynamics in clock gene-deficient mice. *Plos One* **8**, e58884, doi:10.1371/journal.pone.0058884 (2013).
